# Supplementary material for: Defining remission of type 2 diabetes in research studies: A systematic scoping review
Source: PLoS Med. 2020 Oct 28;17(10):e1003396. doi: 10.1371/journal.pmed.1003396 (PMC7592769; doi:10.1371/journal.pmed.1003396)
Supplement: S1 Fig — (PPTX) [file pmed.1003396.s001.pptx]

## Slide 1
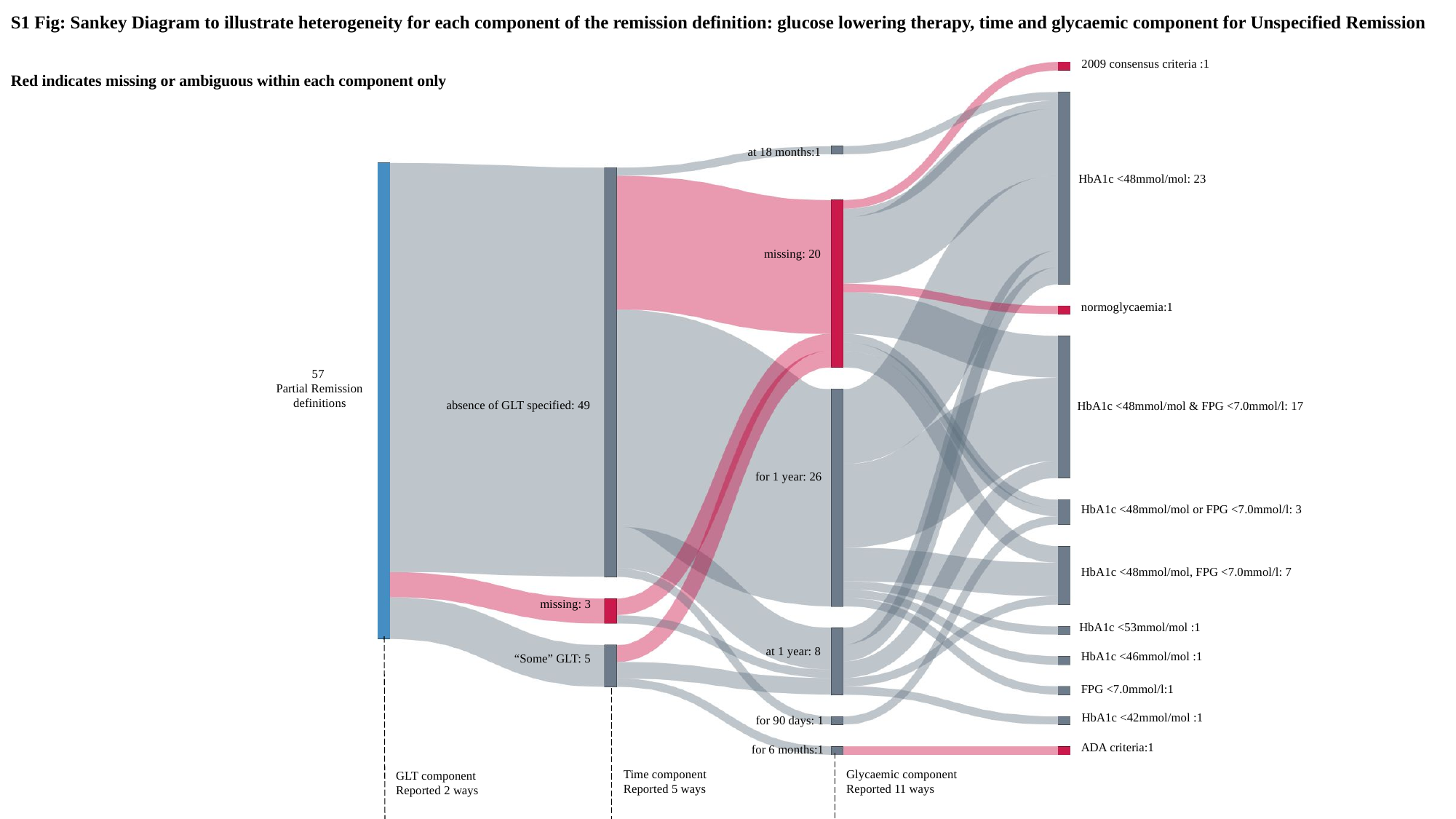

S1 Fig: Sankey Diagram to illustrate heterogeneity for each component of the remission definition: glucose lowering therapy, time and glycaemic component for Unspecified Remission
2009 consensus criteria :1
Red indicates missing or ambiguous within each component only
at 18 months:1
HbA1c <48mmol/mol: 23
missing: 20
normoglycaemia:1
57
Partial Remission definitions
absence of GLT specified: 49
HbA1c <48mmol/mol & FPG <7.0mmol/l: 17
for 1 year: 26
HbA1c <48mmol/mol or FPG <7.0mmol/l: 3
HbA1c <48mmol/mol, FPG <7.0mmol/l: 7
missing: 3
HbA1c <53mmol/mol :1
at 1 year: 8
HbA1c <46mmol/mol :1
“Some” GLT: 5
FPG <7.0mmol/l:1
 HbA1c <42mmol/mol :1
for 90 days: 1
ADA criteria:1
for 6 months:1
Time component
Reported 5 ways
Glycaemic component
Reported 11 ways
GLT component
Reported 2 ways
