# Supplementary material for: Defining remission of type 2 diabetes in research studies: A systematic scoping review
Source: PLoS Med. 2020 Oct 28;17(10):e1003396. doi: 10.1371/journal.pmed.1003396 (PMC7592769; doi:10.1371/journal.pmed.1003396)
Supplement: S2 Fig — (PPTX) [file pmed.1003396.s002.pptx]

## Slide 1
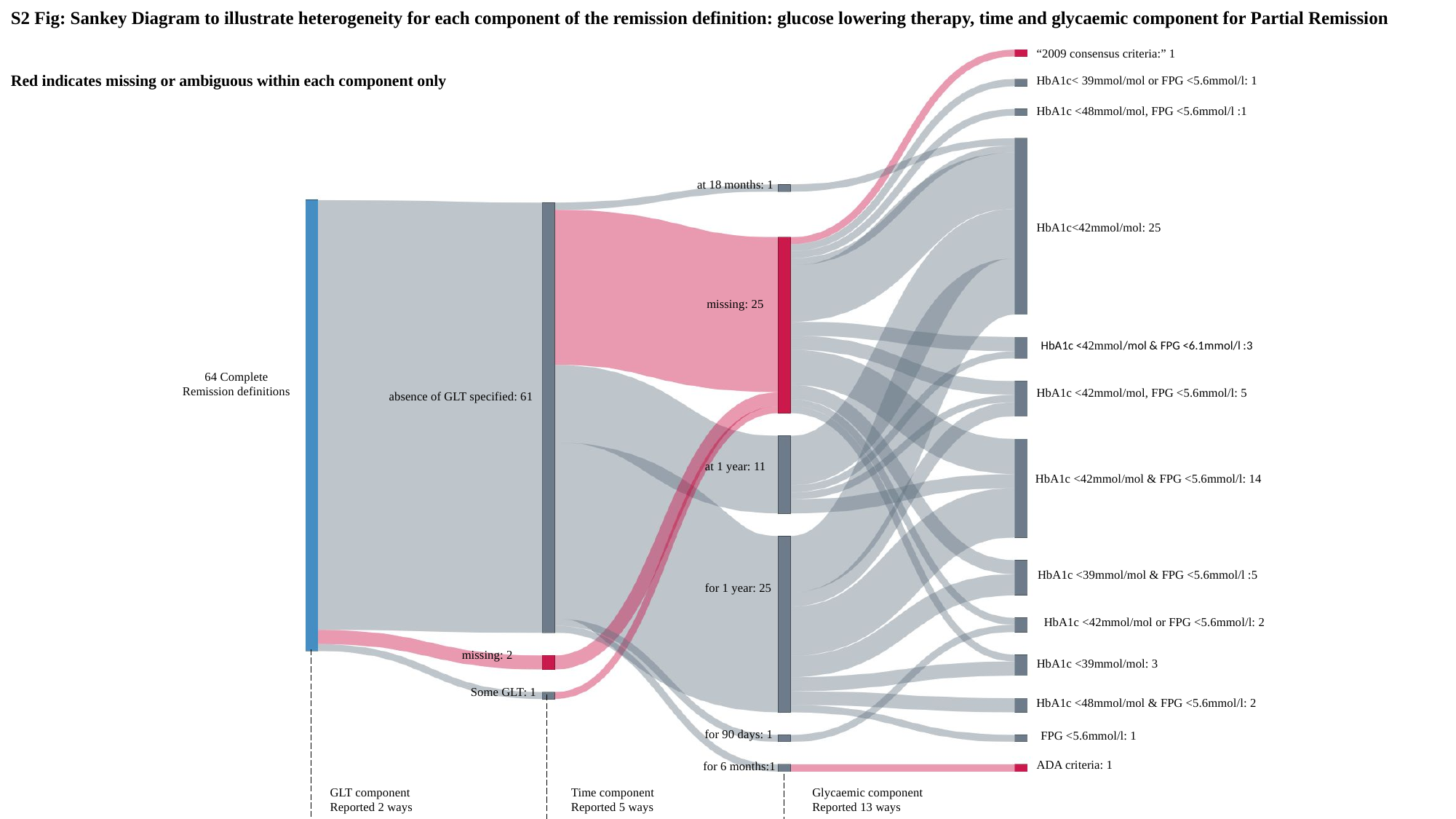

S2 Fig: Sankey Diagram to illustrate heterogeneity for each component of the remission definition: glucose lowering therapy, time and glycaemic component for Partial Remission
“2009 consensus criteria:” 1
HbA1c<42mmol/mol: 25
missing: 25
HbA1c <42mmol/mol & FPG <6.1mmol/l :3
64 Complete Remission definitions
at 1 year: 11
HbA1c <42mmol/mol & FPG <5.6mmol/l: 14
HbA1c <39mmol/mol & FPG <5.6mmol/l :5
for 1 year: 25
missing: 2
HbA1c <39mmol/mol: 3
HbA1c <48mmol/mol & FPG <5.6mmol/l: 2
for 90 days: 1
 FPG <5.6mmol/l: 1
Red indicates missing or ambiguous within each component only
HbA1c< 39mmol/mol or FPG <5.6mmol/l: 1
HbA1c <48mmol/mol, FPG <5.6mmol/l :1
at 18 months: 1
HbA1c <42mmol/mol, FPG <5.6mmol/l: 5
absence of GLT specified: 61
HbA1c <42mmol/mol or FPG <5.6mmol/l: 2
Some GLT: 1
ADA criteria: 1
for 6 months:1
GLT component
Reported 2 ways
Time component
Reported 5 ways
Glycaemic component
Reported 13 ways
