# Supplementary material for: Defining remission of type 2 diabetes in research studies: A systematic scoping review
Source: PLoS Med. 2020 Oct 28;17(10):e1003396. doi: 10.1371/journal.pmed.1003396 (PMC7592769; doi:10.1371/journal.pmed.1003396)
Supplement: S4 Fig — (PPTX) [file pmed.1003396.s004.pptx]

## Slide 1
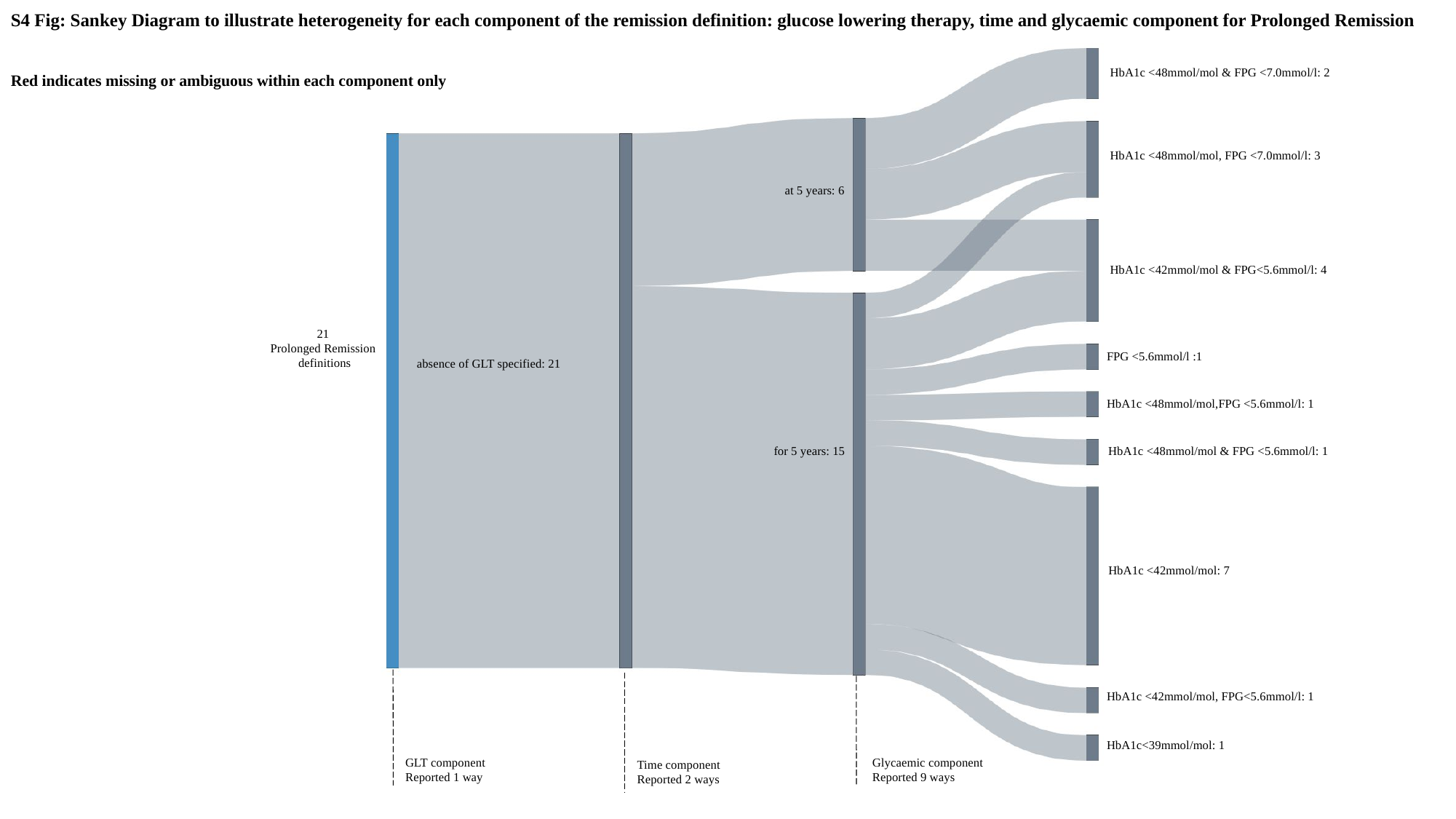

S4 Fig: Sankey Diagram to illustrate heterogeneity for each component of the remission definition: glucose lowering therapy, time and glycaemic component for Prolonged Remission
HbA1c <48mmol/mol & FPG <7.0mmol/l: 2
at 5 years: 6
21
Prolonged Remission
definitions
FPG <5.6mmol/l :1
for 5 years: 15
HbA1c <48mmol/mol & FPG <5.6mmol/l: 1
HbA1c <42mmol/mol: 7
HbA1c <42mmol/mol & FPG<5.6mmol/l: 4
HbA1c<39mmol/mol: 1
Red indicates missing or ambiguous within each component only
HbA1c <48mmol/mol, FPG <7.0mmol/l: 3
absence of GLT specified: 21
HbA1c <48mmol/mol,FPG <5.6mmol/l: 1
HbA1c <42mmol/mol, FPG<5.6mmol/l: 1
GLT component
Reported 1 way
Glycaemic component
Reported 9 ways
Time component
Reported 2 ways
