# Supplementary material for: Defining remission of type 2 diabetes in research studies: A systematic scoping review
Source: PLoS Med. 2020 Oct 28;17(10):e1003396. doi: 10.1371/journal.pmed.1003396 (PMC7592769; doi:10.1371/journal.pmed.1003396)
Supplement: S2 Table — (DOCX) [file pmed.1003396.s006.docx]

**S2 Table PICOS search criteria and sources for the review**

| **PICOS** | **Definition** |
| --- | --- |
| Population | Adults (18 years or over) with type 2 diabetes. |
| Intervention | Surgical, pharmacological or lifestyle interventions measuring remission of type 2 diabetes as an outcome |
| Comparator | Definition of remission will be noted regardless of the comparator or control |
| Outcome | Definition of type 2 diabetes remission |
| Settings | Any settings |
| Study design | Systematic review of observational or interventional primary studies |
| Dates | Database search: November 2009 to 18^th^ July 2020 |
| Databases | MEDLINE, CINAHL, Cochrane Database of Systematic Reviews, EMBASE, |
| Other Exclusions | Papers not published in English  Published before 2009  Studies with less than 100 adult participants with type 2 diabetes available for remission analysis  Conference abstracts, protocols, systematic reviews, reviews, case reports  Previous versions of updated studies with an identical remission definition  Exclude papers focussing on gestational diabetes, T1D, maturity onset diabetes of the young, steroid induced diabetes, prediabetes or impaired glucose tolerance |
