# Supplementary material for: Defining remission of type 2 diabetes in research studies: A systematic scoping review
Source: PLoS Med. 2020 Oct 28;17(10):e1003396. doi: 10.1371/journal.pmed.1003396 (PMC7592769; doi:10.1371/journal.pmed.1003396)
Supplement: S3 Table — (DOCX) [file pmed.1003396.s007.docx]

**S3 Table: Search Strategy: Medline**

| 1 | exp Weight Loss/ |
| --- | --- |
| 2 | exp Diet, Reducing/ |
| 3 | exp Anti-Obesity Agents/ |
| 4 | exp diet/ |
| 5 | exp dietary carbohydrates/ |
| 6 | exp Diet, Ketogenic/ or exp Diet, Vegetarian/ or exp Diet, Gluten-Free/ or exp Diet, Carbohydrate Loading/ or exp Diet, Sodium-Restricted/ or exp Diet, Western/ or exp Diet/ or exp Healthy Diet/ or exp Diet, Paleolithic/ or exp Diet, Fat-Restricted/ or exp Diet, Carbohydrate-Restricted/ or exp Diet, High-Protein/ or exp Diet, Mediterranean/ or exp Diet, Vegan/ or exp Diet, Protein-Restricted/ or exp Diet, High-Fat/ or exp Diet, High-Protein Low-Carbohydrate/ or exp Diet Therapy/ or exp Diet, Diabetic/ |
| 7 | exp Caloric Restriction/ |
| 8 | exp Life Style/ |
| 9 | exp sedentary behavior/ |
| 10 | exp Remission Induction/ |
| 11 | exp Exercise/ |
| 12 | exp Exercise Test/ |
| 13 | exp Exercise therapy/ |
| 14 | ("physical activity" or training or running or jogging or sport or walking or biking or cycling or fitness or "group exercise" or inactivity or "sedentary activity").ti,ab. |
| 15 | fast*.ti,ab. |
| 16 | ("very low calor$ diet" or "VLCD" or "very low energy diet" or "VLED" or "total diet replacement" or "total meal replacement" or "meal replacement").ti,ab. |
| 17 | exp Obesity/ or exp Bariatrics/ or exp Gastroplasty/ or exp Bariatric Surgery/ or exp Obesity, Morbid/ |
| 18 | ((incidence or prevalence) adj3 remission).ti,ab. |
| 19 | GI surgery.ti,ab. |
| 20 | (Gastrectomy or Gastroplasty or gastric laparoscopy or gastric bypass or LAGB or "Roux en Y" or "duodenal switch" or "biliopancreatic diversion" or "jejunoileal band" or "gastric band" or "gastric sleeve").ti,ab. |
| 21 | 1 or 2 or 3 or 4 or 5 or 6 or 7 or 8 or 9 or 10 or 11 or 12 or 13 or 14 or 15 or 16 or 17 or 18 or 19 or 20 |
| 22 | exp Diabetes Mellitus, Type 2/ or exp Diabetes Mellitus/ |
| 23 | (T2D or T2DM or NIDDM or IDDM).ti,ab. |
| 24 | ((diabet* or dm) adj3 II).ti,ab. |
| 25 | ((Diabet* or dm) adj3 two).ti,ab. |
| 26 | exp Insulin Resistance/ |
| 27 | exp obesity, Morbid/ or exp Obesity/ |
| 28 | ((insulin or noninsulin or non-insulin) adj2 (resistan* or depend*)).ti,ab. |
| 29 | 22 or 23 or 24 or 25 or 26 or 27 or 28 |
| 30 | (remission adj4 diabet*).ti,ab |
| 31 | (remission adj4 (T2D or T2DM or NIDDM or IDDM)).ti,ab. |
| 32 | (cure adj4 (diabet* or T2D or T2DM or NIDDM or IDDM)).ti,ab. |
| 33 | (resolv* adj4 (diabet* or T2D or T2DM or NIDDM or IDDM)).ti,ab. |
| 34 | (resolution adj4 (diabet* or T2D or T2DM or NIDDM or IDDM)).ti,ab. |
| 35 | (revers* adj4 (diabet* or T2D or T2DM or NIDDM or IDDM)).ti,ab. |
| 36 | (normoglyc*emic or euglyc&emic).ti,ab. |
| 37 | 30 or 31 or 32 or 33 or 34 or 35 or 36 |
| 38 | 21 and 29 and 37 |
| 39 | limit 38 to humans |
