# Supplementary material for: Defining remission of type 2 diabetes in research studies: A systematic scoping review
Source: PLoS Med. 2020 Oct 28;17(10):e1003396. doi: 10.1371/journal.pmed.1003396 (PMC7592769; doi:10.1371/journal.pmed.1003396)
Supplement: S4 Table — (DOCX) [file pmed.1003396.s008.docx]

**S4 Table: Characteristics of included studies**

|  | **Author** | **Year** | **Study Title** | **Study** | **Country** | **N^a^** | **Intervention/Exposure** | **Guideline Referenced** | **Synonym (alternative/ additional)** |
| --- | --- | --- | --- | --- | --- | --- | --- | --- | --- |
| 1 | Abd Ellatif [65] | 2014 | Long term predictors of success after laparoscopic sleeve gastrectomy | cohort | Egypt | 1089 | Surgery | None | resolution |
| 2 | Abu-Abeid [66] | 2018 | Diabetes resolution after one anastomosis gastric bypass | cohort | Israel | 102 | Surgery | 2009 report (2 definitions) | resolution |
| 3 | Adams [67] | 2012 | Health Benefits of Gastric Bypass Surgery After 6 Years | cohort | USA | 269 | Surgery | None | - |
| 4 | Ahuja [48] | 2018 | Predicting remission of diabetes post metabolic surgery: A comparison of ABCD, diarem, and DRS scores | cohort | India | 102 | Surgery | 2009 report (3 definitions) | resolution |
| 5 | Alhambra-Exposito [39] | 2017 | Variations in diabetes resmission rates after bariatric surgery in Spanish adults according to the use of different diagnostic criteria for diabetes | cohort | Spain | 127 | Surgery | 1. Rubio MA, Monereo S, Lecube A, Resa J, Masdevall C, de la Cruz VF, et al. Joint position statement of the SEEN-SECO-SEEDO-SED societies on metabolic surgery for type 2 diabetes mellitus. Endocrinol Nutr. 2013;60(10):547–8.  2. American Diabetes Association. Diagnosis and classification of diabetes mellitus. Standard of medical care of diabetes −2017. Diabetes Care. 2017;40 (Suppl 1):S1–2. | - |
| 6 | Al-Khyatt [68] | 2016 | Laparoscopic Roux en-Y Gastric Bypass Using a Modified Retrocolic–Supracolic Approach: Outcomes from 300 Patients | cohort | UK | 110 | Surgery | None | resolution |
| 7 | Almalki [69] | 2018 | Laparoscopic gastric bypass for the treatment of type 2 diabetes: a comparison of Roux-en-Y versus single anastomosis gastric bypass | cohort | Taiwan | 406 | Surgery | 2009 report (2 definitions) | - |
| 8 | Al-Sabah [70] | 2014 | Remission of Type 2 Diabetes Mellitus after Laparoscopic Sleeve Gastrectomy | cohort | Kuwait | 107 | Surgery | None | cure |
| 9 | Aminian [71] | 2020 | Late Relapse of Diabetes after Bariatric Surgery: Not Rare, but Not a Failure | cohort | USA | 736 | Surgery | 1. Brethauer SA, Aminian A, Romero-Talamas H, Batayyah E, Mackey J, Kennedy L, et al. Can diabetes be surgically cured? Long-term metabolic effects of bariatric surgery in obese patients with type 2 diabetes mellitus. Ann Surg. 2013; 258:628–636.  2. Brethauer SA, Kim J, el Chaar M, Papasavas P, Eisenberg D, Rogers A et al. Standardized outcomes reporting in metabolic and bariatric surgery. Surg Obes Relat Dis. 2015;11(3):489–506.  3. Aminian A, Brethauer SA, Andalib A, Punchai S, Mackey J, Rodriguez J et al. Can Sleeve Gastrectomy “Cure” Diabetes? Long-term Metabolic Effects of Sleeve Gastrectomy in Patients with Type 2 Diabetes. Ann Surg. 2016; 264: 674-681. | - |
| 10 | Aminian [72] | 2017 | Individualized Metabolic Surgery Score: Procedure Selection Based on Diabetes Severity | cohort | USA | 900 | Surgery | 2009 report (2 definitions) | - |
| 11 | Aminian [73] | 2016 | Can Sleeve Gastrectomy “Cure” Diabetes? Long-term Metabolic Effects of Sleeve Gastrectomy in Patients With Type 2 Diabetes | cohort | USA | 134 | Surgery | 1. 2009 report (3 definitions) 2. Brethauer SA, Aminian A, Romero-Talamas H, Batayyah E, Mackey J, Kennedy L, et al. Can diabetes be surgically cured? Long-term metabolic effects of bariatric surgery in obese patients with type 2 diabetes mellitus. Ann Surg. 2013; 258:628–636.  3.Brethauer SA, Kim J, el Chaar M, Papasavas P, Eisenberg D, Rogers A et al. Standardized outcomes reporting in metabolic and bariatric surgery. Surg Obes Relat Dis. 2015;11(3):489–506. | cure |
| 12 | Aminian [49] | 2014 | Risk prediction of complications of metabolic syndrome before and 6 years after gastric bypass | cohort | USA | 131 | Surgery | Brethauer SA, Aminian A, Romero-Talamas H, Batayyah E, Mackey J, Kennedy L, et al. Can diabetes be surgically cured? Long-term metabolic effects of bariatric surgery in obese patients with type 2 diabetes mellitus. Ann Surg. 2013; 258:628–636. | - |
| 13 | Araia [50] | 2014 | Resolution of Diabetes After Bariatric Surgery Among Predominantly African-American Patients Race has no Effect in Remission of Diabetes After Bariatric Surgery | cohort | USA | 119 | Surgery | 2009 report (1 definition) | resolution |
| 14 | Ardestani [74] | 2015 | Insulin Cessation and Diabetes Remission After Bariatric Surgery in Adults With Insulin-Treated Type 2 Diabetes | cohort | USA | 5225 | Surgery | DeMaria EJ, Winegar DA, Pate VW, Hutcher NE, Ponce J, Pories WJ. Early postoperative outcomes of metabolic surgery to treat diabetes from sites participating in the ASMBS bariatric surgery center of excellence program as reported in the Bariatric Outcomes Longitudinal Database. Ann Surg. 2010; 252:559–566 | resolution |
| 15 | Aron-Wisnewsky [51] | 2017 | The advanced-DiaRem score improves prediction of diabetes remission 1 year post-Roux-en-Y gastric bypass | cohort | France, Israel | 213 | Surgery | 2009 report (2 definitions) | - |
| 16 | Arterburn [34] | 2013 | Comparative Effectiveness of Bariatric Surgery versus Nonsurgical Treatment of Type 2 Diabetes among Severely Obese Adults | cohort | USA | 63622 | Surgery vs usual care | None | resolution |
| 17 | Arterburn [35] | 2013 | A multisite study of long-term remission and relapse of type 2 diabetes mellitus following gastric bypass | cohort | USA | 4434 | Surgery | 2009 report (2 definitions) | resolution, cure |
| 18 | Aung [75] | 2016 | Bariatric Surgery for Patients With Early-Onset vs Late-Onset Type 2 Diabetes | cohort | Taiwan | 558 | Surgery | 2009 report (3 definitions) | - |
| 19 | Bayham [76] | 2012 | Early Resolution of Type 2 Diabetes Seen After Roux-en-Y Gastric Bypass and Vertical Sleeve Gastrectomy | cohort | USA | 109 | Surgery | None | resolution |
| 20 | Behbehani [77] | 2014 | Metabolic outcomes 2 years following gastric bypass surgery in people with type 2 diabetes: an observational cohort study | cohort | UK | 101 | Surgery | 2009 report (2 definitions) | resolution |
| 21 | Bhasker [78] | 2018 | Selection of Bypass vs Sleeve for the Management of Type-2 Diabetes in Severely Obese: Could Ethnicity Play a Role? | cohort | India | 186 | Surgery | None | - |
| 22 | Bhasker [25] | 2015 | Predictors of Remission of T2DM and Metabolic Effects after Laparoscopic Roux-en-y Gastric Bypass in Obese Indian Diabetics—a 5-Year Study | cohort | India | 106 | Surgery | “ADA criteria” | resolution |
| 23 | Biertho [79] | 2014 | Laparoscopic Sleeve Gastrectomy: With or without Duodenal Switch? A Consecutive Series of 800 Cases | cohort | Canada | 193 | Surgery | None | resolution |
| 24 | Blackstone [41] | 2012 | Type 2 diabetes after gastric bypass: Remission in five models using HbA1c, fasting blood glucose, and medication status | cohort | USA | 505 | Surgery | 2009 report (5 definitions) | - |
| 25 | Bohula [36] | 2018 | Effect of lorcaserin on prevention and remission of type 2 diabetes in overweight and obese patients (CAMELLIA-TIMI 61): a randomised, placebo-controlled trial | RCT | USA | 6816 | Pharmacological | None | Persistent remission of hyper-glycaemia, sustained remission of hyper-glycaemia, any remission of hyper-glycaemia, persistent normo-glycaemia, sustained normo-glycaemia, any normo-glycaemia |
| 26 | Boza [52] | 2014 | Metabolic Surgery: Roux-en-Y Gastric Bypass and Variables Associated with Diabetes Remission in Patients with BMI <35 | cohort | Chile | 100 | Surgery | American Diabetes Association. Standards of medical care in diabetes—2012. Diabetes Care. 2012;35 Suppl 1: S11–63. | resolution |
| 27 | Brethauer [80] | 2013 | Can Diabetes Be Surgically Cured? Long-Term Metabolic Effects of Bariatric Surgery in Obese Patients with Type 2 Diabetes Mellitus | cohort | USA | 217 | Surgery | 1. 2009 report (3 definitions) 2. Schauer PR, Burguera B, Ikramuddin S, Cottam D, Gourash W, Hamad G, et al. Effect of laparoscopic Roux-en Y gastric bypass on type 2 diabetes mellitus. Ann Surg. 2003; 238: 467–484. | cure |
| 28 | Bruno [81] | 2015 | What is the impact of sleeve gastrectomy and gastric bypass on metabolic control of diabetes? A clinic-based cohort of Mediterranean diabetic patients | cohort | Italy | 135 | Surgery | 2009 report (1 definition) | - |
| 29 | Camerini [82] | 2016 | The long-term impact of biliopancreatic diversion on glycemic control in the severely obese with type 2 diabetes mellitus in relation to preoperative duration of diabetes | cohort | Italy | 120 | Surgery | None | resolution |
| 30 | Chen [83] | 2012 | Attitudes Toward Diabetes Affect Maintenance of Drug-Free Remission in Patients With Newly Diagnosed Type 2 Diabetes After Short-Term Continuous Subcutaneous Insulin Infusion Treatment | cohort | China | 158 | Pharmacological | None | - |
| 31 | Chen [53] | 2018 | Prediction of type 2 diabetes remission after metabolic surgery: a comparison of the individualized metabolic surgery score and the ABCD score | cohort | Taiwan | 310 | Surgery | 2009 report (1 definition) | - |
| 32 | Chen [84] | 2016 | Gastric bypass surgery leads to long-term remission or improvement of type 2 diabetes and significant decrease of microvascular and macrovascular complications | cohort | USA | 173 | Surgery | None | resolution |
| 33 | Chikunguwo [85] | 2010 | Analysis of factors associated with durable remission of diabetes after Roux-en-Y gastric bypass | cohort | USA | 177 | surgery | None | resolution |
| 34 | Cottam [86] | 2018 | An Analysis of Mid-Term Complications, Weight Loss, and Type 2 Diabetes Resolution of Stomach Intestinal Pylorus-Sparing Surgery (SIPS) Versus Roux-En-Y Gastric Bypass (RYGB) with Three-Year Follow-Up | cohort | USA | 186 | Surgery | None | resolution |
| 35 | Courcoulas [87] | 2018 | Seven-Year Weight Trajectories and Health Outcomes in the Longitudinal Assessment of Bariatric Surgery (LABS) Study | cohort | USA | 747 | Surgery | None | resolution |
| 36 | Craig Wood [54] | 2018 | Performance of the DiaRem Score for Predicting Diabetes Remission in Two Health Systems Following Bariatric Surgery Procedures in Hispanic and non-Hispanic White Patients | cohort | USA | 491 | Surgery | Buchwald H, Estok R, Fahrbach K, Banel D, Jensen MD, Pories WJ, et al. Weight and type 2 diabetes after bariatric surgery: systematic review and meta-analysis. Am J Med. 2009;122(3):248–56. | - |
| 37 | Dambha-Miller [88] | 2020 | Behaviour change, weight loss and remission of Type 2 Diabetes: a community-based prospective cohort study | cohort | UK | 730 | Lifestyle | None | - |
| 38 | Dang [89] | 2019 | Predictive factors for diabetes remission after bariatric surgery | cohort | Canada | 207 | Surgery | None | - |
| 39 | Davies [90] | 2014 | Long-Term Diabetic Response to Gastric Bypass | cohort | USA | 707 | Surgery | None | resolution |
| 40 | Debedat [91] | 2018 | Long-term Relapse of Type 2 Diabetes After Roux-en-Y Gastric Bypass: Prediction and Clinical Relevance | cohort | France, Italy, Germany | 175 | Surgery | 2009 report (1 definition) | - |
| 41 | de Oliveira [92] | 2018 | Predictors of Long-Term Remission and Relapse of Type 2 Diabetes Mellitus Following Gastric Bypass in Severely Obese Patients | cohort | Brazil | 254 | Surgery | 2009 report (2 definitions) | - |
| 42 | Dicker [93] | 2019 | Prediction of Long-Term Diabetes Remission After RYGB, Sleeve Gastrectomy, and Adjustable Gastric Banding Using DiaRem and Advanced-DiaRem Scores | cohort | Israel | 1459 | Surgery | 2009 report (2 definitions) | resolution |
| 43 | Dicker [26] | 2016 | Long-Term Outcomes of Three Types of Bariatric Surgery on Obesity and Type 2 Diabetes Control and Remission | cohort | Israel | 1685 | Surgery | None | resolution |
| 44 | Dixon [94] | 2013 | Predicting the glycemic response to gastric bypass surgery in patients with type 2 diabetes | cohort | Taiwan | 154 | Surgery | None | - |
| 45 | Dorman [95] | 2012 | Case-Matched Outcomes in Bariatric Surgery for Treatment of Type 2 Diabetes in the Morbidly Obese Patient | cohort | USA | 172 | Surgery & pharmacological | None | resolution |
| 46 | Douglas [33] | 2015 | Bariatric Surgery in the United Kingdom: A Cohort Study of Weight Loss and Clinical Outcomes in Routine Clinical Care | cohort | UK | 2616 | Surgery | None | resolution |
| 47 | Du [96] | 2018 | Effects of Laparoscopic Roux-en-Y Gastric Bypass on Chinese Type 2 Diabetes Mellitus Patients with Different Levels of Obesity: Outcomes After 3 Years' Follow-Up | cohort | China | 103 | Surgery | 2009 report (1 definition) | resolution |
| 48 | Durmush [97] | 2014 | Short-term outcomes of sleeve gastrectomy for morbid obesity: Does staple line reinforcement matter? | cohort | Australia | 128 | Surgery | None | resolution |
| 49 | Egan [98] | 2016 | The Impact of Laparoscopic Adjustable Gastric Banding on an NHS Cohort of Type 2 Diabetics: a Prospective Cohort Study | cohort | UK | 120 | Surgery | None | - |
| 50 | English [99] | 2015 | Predicting remission of diabetes after RYGB surgery following intensive management to optimize preoperative glucose control | cohort | USA | 245 | Surgery | None | resolution |
| 51 | Esposito [100] | 2014 | The effects of a Mediterranean diet on the need for diabetes drugs and remission of newly diagnosed type 2 diabetes: Follow-up of a randomized trial | RCT | Italy | 215 | Dietary | None | - |
| 52 | Finno [101] | 2020 | Single Versus Double-Anastomosis Duodenal Switch: Single-Site Comparative Cohort Study in 440 Consecutive Patients | cohort | Spain | 157 | Surgery | None | resolution |
| 53 | Friedman [29] | 2019 | The association Between Kidney Disease and Diabetes Remission in Bariatric Surgery Patients With Type 2 Diabetes | cohort | USA | 737 | Surgery | 2009 report (2 definitions) | - |
| 54 | Girundi [102] | 2016 | Type 2 Diabetes Mellitus remission eighteen months after Roux-en-Y gastric bypass | cohort | Brazil | 468 | Surgery | Sociedade Brasileira de Diabetes (SBD). Algoritmo para o tratamento do diabetes tipo 2 -atualização 2011.Posicionamento oficial SBD número-2011. | resolution |
| 55 | Gregg [14] | 2012 | Association of an intensive lifestyle intervention with remission of type 2 diabetes | cohort | USA | 4503 | Lifestyle | 2009 report (2 definitions) | cure |
| 56 | Guerreiro [103] | 2019 | Long-Term Weight Loss and Metabolic Sundrome Remission after Bariatric Surgery: The Effect of Sex, Age, Metabolic Parameters and Surgical Technique- A 4-Year Follow-Up Study | cohort | Portugal | 272 | Surgery | None | resolution |
| 57 | Gullick [104] | 2015 | Association of Race and Socioeconomic Status with Outcomes Following Laparoscopic Roux-en-Y Gastric Bypass | cohort | USA | 254 | Surgery | None | - |
| 58 | Gulliford [105] | 2016 | Effect of Contemporary Bariatric Surgical Procedures on Type 2 Diabetes Remission. A Population-Based Matched Cohort Study | cohort | UK | 1652 | Surgery | None | - |
| 59 | Hall [106] | 2010 | Preoperative Factors Predicting Remission of Type 2 Diabetes Mellitus After Roux-en-Y Gastric Bypass Surgery for Obesity | cohort | UK | 110 | Surgery | 2009 report (1 definition) | resolution |
| 60 | Haider [107] | 2020 | Remission of type 2 diabetes following long-term treatment with injectable testosterone undecanoate in patients with hypogonadism and type 2 diabetes: 11-year data from a real-world registry study | cohort | Germany | 356 | Pharmacological | None | - |
| 61 | Hariri [108] | 2017 | Preoperative insulin therapy as a marker for type 2 diabetes remission in obese patients after bariatric surgery | cohort | USA | 180 | Surgery | Brethauer SA, Kim J, el Chaar M, Papasavas P, Eisenberg D, Rogers A et al. Standardized outcomes reporting in metabolic and bariatric surgery. Surg Obes Relat Dis. 2015;11(3):489–506. | resolution |
| 62 | Haruta [109] | 2017 | Long-Term Outcomes of Bariatric and Metabolic Surgery in Japan: Results of a Multi-Institutional Survey | cohort | Japan | 136 | Surgery | Brethauer SA, Kim J, el Chaar M, Papasavas P, Eisenberg D, Rogers A et al. Standardized outcomes reporting in metabolic and bariatric surgery. Surg Obes Relat Dis. 2015;11(3):489–506. | - |
| 63 | Hatoum [110] | 2016 | Clinical factors associated with remission of obesity-related comorbidities after bariatric surgery | cohort | USA | 4848 | Surgery | None | resolution |
| 64 | Hayes M [111] | 2011 | A Model for Predicting the Resolution of Type 2 Diabetes in Severely Obese Subjects Following Roux-en Y Gastric Bypass Surgery | cohort | NZ | 127 | Surgery | None | resolution |
| 65 | Hayes S [55] | 2015 | The effect of insurance status on pre- and post-operative bariatric surgery outcomes | cohort | USA | 684 | Surgery | None | - |
| 66 | Hoerger [112] | 2010 | Cost-effectiveness of bariatric surgery for severely obese adults with diabetes | cost-effectiveness model | USA | - | Surgery | None | - |
| 67 | Hofsø [113] | 2019 | Gastric bypass versus sleeve gastrectomy in patients with type 2 diabetes (Osebery): a single-centre, triple-blind, randomised controlled trial | RCT | Norway | 107 | Surgery | 2009 report (1 definition) | - |
| 68 | Honarmand [114] | 2017 | Type 2 diabetes remission rates 1-year post-Roux-en-Y gastric bypass and validation of the DiaRem score: the Ontario Bariatric Network experience | cohort | Canada | 900 | Surgery | American Diabetes Association. Classification and diagnosis of diabetes. Sec. 2. Diabetes Care 2015; 38(Suppl. 1): S8–S16. | - |
| 69 | Hsu [115] | 2015 | Effect of Bariatric Surgery vs Medical Treatment on Type 2 Diabetes in Patients With Body Mass Index Lower Than 35Five-Year Outcomes | RCT | Taiwan | 300 | Surgery & pharmacological | 2009 report (2 definitions) | - |
| 70 | Hussain [116] | 2019 | Short- and Mid-term Outcomes of 527 One Anastomosis Gastric Bypass/Mini-Gastric Bypass (OAGB/MGB) Operations: Retrospective Study | cohort | UK | 124 | Surgery | None | resolution |
| 71 | Iacobellis [117] | 2015 | Predictors of Short-Term Diabetes Remission After Laparoscopic Roux-en-Y Gastric Bypass | cohort | USA | 206 | Surgery | 2009 report (1 definition) | resolution |
| 72 | Ikramuddin [118] | 2016 | Durability of Addition of Roux-en-Y Gastric Bypass to Lifestyle Intervention and Medical Management in Achieving Primary Treatment Goals for Uncontrolled Type 2 Diabetes in Mild to Moderate Obesity: A Randomized Control Trial | RCT | USA & Taiwan | 120 | Surgery | 2009 report (2 definitions) | - |
| 73 | Inabnet [119] | 2012 | Early Outcomes of Bariatric Surgery in Patients with Metabolic Syndrome: An Analysis of the Bariatric Outcomes Longitudinal Database | cohort | USA | 6103 | Surgery | None | resolution |
| 74 | Jakobsen [120] | 2018 | Association of Bariatric Surgery vs Medical Obesity Treatment With Long-term Medical Complications and Obesity-Related Comorbidities | cohort | Norway | 491 | Surgery | None | - |
| 75 | Jans [121] | 2019 | Duration of type 2 diabetes and remission rates after bariatric surgery in Sweden 2007-2015: A registry-based cohort study | cohort | Sweden | 3594 | Surgery | Brethauer SA, Kim J, el Chaar M, Papasavas P, Eisenberg D, Rogers A et al. Standardized outcomes reporting in metabolic and bariatric surgery. Surg Obes Relat Dis. 2015;11(3):489–506. | - |
| 76 | Jimenez [122] | 2012 | Long-term effects of sleeve gastrectomy and roux-en-y gastric bypass surgery on type 2 diabetes mellitus in morbidly obese subjects | cohort | Spain | 153 | Surgery | 2009 report (1 definition) | resolution |
| 77 | Jimenez [123] | 2015 | Remission of type 2 diabetes after roux-en-y gastric bypass or sleeve gastrectomy is associated with a distinct glycemic profile | cohort | Spain | 232 | Surgery | 2009 report (1 definition) | - |
| 78 | Jönsson [124] | 2017 | Diabetes Resolution and Work Absenteeism After Gastric Bypass: a 6-Year Study | cohort | Sweden | 656 | Surgery | None | resolution |
| 79 | Karter [21] | 2014 | Incidence of Remission in Adults With Type 2 Diabetes: The Diabetes & Aging Study | cohort | USA | 122781 | - | 2009 report (3 definitions) | - |
| 80 | Kaska [125] | 2014 | Dynamics of type 2 diabetes mellitus laboratory remission after Roux-en-Y gastric bypass in patients with body mass index lower than 35 kg/m2 and higher than 35 kg/m2 in a 3-year observation period | cohort | Poland | 112 | Surgery | 2009 report (1 definition) | regression, cure |
| 81 | Khalaj [126] | 2020 | Comparing the Efficacy and Safety of Roux-en-Y Gastric Bypass with One-Anastomosis Gastric Bypass with a Biliopancreatic Limb of 200 or 160 cm: 1-Year Results of the Tehran Obesity Treatment Study (TOTS) | cohort | Iran | 294 | Surgery | None | - |
| 82 | Kim JW [127] | 2012 | Outcome after gastrectomy in gastric cancer patients with type 2 diabetes | cohort | S Korea | 403 | Surgery | None | resolution |
| 83 | Kim S [128] | 2010 | Long-term follow-up of the metabolic profiles in obese patients with type 2 diabetes mellitus after roux-en-Y gastric bypass | cohort | S korea | 219 | Surgery | American Diabetes Association. Standards of medical care in diabetes—2008. Diabetes Care. 2008;31(suppl 1):S12–S54. | resolution |
| 84 | Kothari [129] | 2017 | Long-term (>10-year) outcomes after laparoscopic Roux-en-Y gastric bypass | cohort | USA | 367 | Surgery | None | resolution |
| 85 | Kular [27] | 2015 | Seven Years of Mini-Gastric Bypass in Type II Diabetes Patients with a Body Mass Index <35 kg/m(2) | cohort | India | 983 | Surgery | 2009 report (2 definitions) | resolution |
| 86 | Lager [130] | 2018 | Metabolic Parameters, Weight Loss, and Comorbidities 4 Years After Roux-en-Y Gastric Bypass and Sleeve Gastrectomy | cohort | USA | 231 | Surgery | 2009 report (1 definition) | - |
| 87 | Lean [131] | 2019 | Durability of a primary care-led weight-management intervention for remission of type 2 diabetes: 2-year results of the DiRECT open label, cluster-randomised trial | CRT | UK | 256 | Lifestyle | McCombie L, Leslie W, Taylor R, Kennon B, Sattar N, Lean MEJ. Beating type 2 diabetes into remission. BMJ. 2017; 358 | - |
| 88 | Lean [13] | 2018 | Primary care-led weight management for remission of type 2 diabetes (DiRECT): an open-label, cluster-randomised trial | CRT | UK | 290 | Lifestyle | None | - |
| 89 | Lee MH [132] | 2015 | Predictors of Long-Term Diabetes Remission After Metabolic Surgery | cohort | Taiwan | 157 | Surgery | 2009 report (3 definitions) | - |
| 90 | Lee PC [133] | 2018 | Ethnicity Does Not Influence Glycemic Outcomes or Diabetes Remission After Sleeve Gastrectomy or Gastric Bypass in a Multiethnic Asian Cohort | cohort | Singapore | 145 | Surgery | 2009 report (1 definition) | - |
| 91 | Lee SK [47] | 2016 | Roux-en-Y Gastric Bypass vs. Sleeve Gastrectomy vs. Gastric Banding: The First Multicenter Retrospective Comparative Cohort Study in Obese Korean Patients | cohort | S Korea | 102 | Surgery | None | resolution |
| 92 | Lee W [134] | 2012 | Comparative study of diabetes mellitus resolution according to reconstruction type after gastrectomy in gastric cancer patients with diabetes mellitus | cohort | S Korea | 229 | Surgery | 2009 report (3 definitions) | resolution |
| 93 | Lee WJ [135] | 2015 | The Effect and Predictive Score of Gastric Bypass and Sleeve Gastrectomy on Type 2 Diabetes Mellitus Patients with BMI < 30 kg/m(2) | cohort | Taiwan | 512 | Surgery | 2009 report (2 definitions) | -- |
| 94 | Lee WJ [136] | 2016 | Bariatric versus diabetes surgery after five years of follow up | cohort | Taiwan | 618 | Surgery | 2009 report (2 definitions) |  |
| 95 | Lee WJ [137] | 2017 | Metabolic Surgery for Diabetes Treatment: Sleeve Gastrectomy or Gastric Bypass? | cohort | Taiwan | 579 | Surgery | 2009 report (2 definitions) | resolution |
| 96 | Lee WJ [138] | 2016 | Preoperative Prediction of Type 2 Diabetes Remission After Gastric Bypass Surgery: a Comparison of DiaRem Scores and ABCD Scores | cohort | Taiwan | 245 | Surgery | 2009 report (3 definitions) | resolution |
| 97 | Lee WJ [56] | 2012 | C-peptide Predicts the Remission of Type 2 Diabetes After Bariatric Surgery | cohort | Taiwan | 205 | Surgery | None | resolution |
| 98 | Lee WJ [139] | 2013 | Predicting success of metabolic surgery: age, body mass index, C-peptide, and duration score | cohort | Taiwan | 239 | Surgery | 2009 report (1 definition) | - |
| 99 | Lemus [140] | 2018 | The impact of bariatric surgery on insulin-treated type 2 diabetes patients | cohort | Canada | 2047 | Surgery | 1. 2009 report (2 definitions) 2. Rubino F, Nathan DM, Eckel RH, Schauer PR, Alberti KG, Zimmet PZ, et al. Metabolic surgery in the treatment algorithm for type 2 diabetes: a joint statement by International Diabetes Organizations. Diabetes Care. 2016; 39(6):861–877. | resolution |
| 100 | Liang H [141] | 2018 | The predictive factors for diabetic remission in Chinese patients with BMI > 30kg/m2 and BMI < 30kg/m2 are different | cohort | China | 144 | Surgery | None | - |
| 101 | Liang Z [142] | 2013 | Effect of laparoscopic Roux-en-Y gastric bypass surgery on type 2 diabetes mellitus with hypertension: A randomized controlled trial | RCT | China | 101 | Surgery & pharmacological | No definition | - |
| 102 | Liu [143] | 2013 | Fasting plasma glucose after intensive insulin therapy predicted long-term glycemic control in newly diagnosed type 2 diabetic patients | cohort | China | 188 | Pharmacological | None | - |
| 103 | Liu [144] | 2015 | Insulin requirement profiles of short-term intensive insulin therapy in patients with newly diagnosed type 2 diabetes and its association with long-term glycemic remission | RCT | China | 100 | Pharmacological & lifestyle | Li Y, Xu W, Liao Z, Yao B, Chen X, Huang Z, et al. Induction of Long-term Glycemic Control in Newly Diagnosed Type 2 Diabetic Patients Is Associated With Improvement of β-Cell Function. Diabetes Care. 2004; 11: 2597-602. | - |
| 104 | Madsen [23] | 2019 | Effect of Roux-en-Y gastric bypass surgery on diabetes remission and complications in individuals with type 2 diabetes: a Danish population-based matched cohort study | cohort | Denmark | 2185 | Surgery | 2009 report (1 definition) | resolution |
| 105 | Mathew [145] | 2015 | Effect of metabolic surgery on type 2 diabetes remission: A matched group analysis | cohort | India | 305 | Surgery | None | - |
| 106 | Mathew [146] | 2015 | Metabolic effects of three different bariatric procedures-a retrospective study | cohort | India | 176 | Surgery | None | - |
| 107 | McTigue [37] | 2020 | Comparing the 5-year Diabetes Outcomes of Sleeve Gastrectomy and Gstric Bypass The National patient-Centred Clnical Research Network (PCORNet) Bariatric Study | cohort | USA | 9710 | Surgery | 2009 report (1 definition) | - |
| 108 | Moh [28] | 2020 | Matabolic Surgery Diabetes Remission (MDR) Score: a New Preoperative Scoring System for Predicting Type 2 Diabetes Remission at 1 Year After Metabolic Surgery in the Singapore Multi-ethnic Asian setting | cohort | Singapore | 114 | Surgery | Lee WJ, Almulaifi A, Tsou JJ, Ser KH, Lee YC, Chen SC. Laparoscopic sleeve gastrectomy for type 2 diabetes mellitus: predicting the success by abcd score. Surg Obes Relat Dis. 2015; 11:991–6. | - |
| 109 | Mu [147] | 2012 | Effects of a combination of oral anti‐diabetes drugs with basal insulin therapy on β‐cell function and glycaemic control in patients with newly diagnosed type 2 diabetes | RCT | China | 125 | Pharmacological | None | -- |
| 110 | Murphy [42] | 2018 | Laparoscopic Sleeve Gastrectomy Versus Banded Roux-en-Y Gastric Bypass for Diabetes and Obesity: a Prospective Randomised Double-Blind Trial | RCT | NZ | 109 | Surgery | None | - |
| 111 | Musella [148] | 2014 | The laparoscopic mini-gastric bypass: The Italian experience: Outcomes from 974 consecutive cases in a multicenter review | cohort | Italy | 201 | Surgery | None (1 definition) | resolution |
| 112 | Musella [149] | 2016 | Efficacy of Bariatric Surgery in Type 2 Diabetes Mellitus Remission: the Role of Mini Gastric Bypass/One Anastomosis Gastric Bypass and Sleeve Gastrectomy at 1 Year of Follow-up. A European survey | cohort | Italy, Germany, Netherlands, Portugal and Czech Republic | 206 | Surgery | 2009 report | - |
| 113 | Naitoh [150] | 2018 | Efficacy of Sleeve Gastrectomy with Duodenal-Jejunal Bypass for the Treatment of Obese Severe Diabetes Patients in Japan: a Retrospective Multicenter Study | cohort | Japan | 298 | Surgery | Brethauer SA, Kim J, el Chaar M, Papasavas P, Eisenberg D, Rogers A et al. Standardized outcomes reporting in metabolic and bariatric surgery. Surg Obes Relat Dis. 2015;11(3):489–506. | resolution |
| 114 | Ng [151] | 2015 | Ethnic variation in weight loss, but not co-morbidity remission, after laparoscopic gastric banding and Roux-en-Y gastric bypass | cohort | USA | 500 | Surgery | None | - |
| 115 | Nor Hanipah [152] | 2019 | Laparoscopic loop duodenaljejunal bypass with sleeve gastrectomy in type 2 diabetic patients | cohort | Taiwan | 163 | Surgery | 2009 report (2 definitions) | resolution |
| 116 | Nora [31] | 2017 | Should Roux-en-Y gastric bypass biliopancreatic limb length be tailored to achieve improved diabetes outcomes? | cohort | Portugal | 114 | Surgery | None | - |
| 117 | O'Rourke [153] | 2019 | Serum biomarkers of inflammation and adiposity in the LABS cohort: associations with metabolic disease and surgical outcomes | cohort | USA | 352 | Surgery | None | - |
| 118 | Panunzi [154] | 2016 | Determinants of Diabetes Remission and Glycemic Control after Bariatric Surgery | cohort | Sweden, Italy, Australia | 624 | Surgery | None | - |
| 119 | Park [155] | 2016 | Prediction of Diabetes Remission in Morbidly Obese Patients After Roux-en-Y Gastric Bypass | cohort | South Korea | 102 | Surgery | 2009 report (2 definitions) | resolution |
| 120 | Park [156] | 2016 | Laparoscopic Roux-en-Y gastric bypass in obese Korean patients: efficacy and potential adverse events | cohort | South Korea | 104 | Surgery | None | resolution |
| 121 | Pereyra-Garcia Castro [40] | 2019 | Efficacy in type 2 diabetes mellitus remission in patients undergoing bariatric surgery | cohort | Spain | 106 | Surgery | 1. 2009 report (3 definitions) 2. Rubio MA, Monereo S, Lecube A, Resa J, Masdevall C, de la Cruz VF, et al. Joint position statement of the SEEN-SECO-SEEDO-SED societies on metabolic surgery for type 2 diabetes mellitus. Endocrinol Nutr. 2013;60(10):547–8. | - |
| 122 | Pessoa [157] | 2020 | Factors Mediating Type 2 Diabetes Remission and Relapse after Gastric Bypass Surgery | cohort | USA | 621 | Surgery | 1. 2009 report (1 definition) 2. Brethauer SA, Kim J, el Chaar M, Papasavas P, Eisenberg D, Rogers A et al. Standardized outcomes reporting in metabolic and bariatric surgery. Surg Obes Relat Dis. 2015;11(3):489–506. 3.. Madsen LR, Baggesen LM, Richelsen B, Thomsen RW. Effect of Roux-en-Y gastric bypass surgery on diabetes remission and complications in individuals with type 2 diabetes: a Danish population-based matched cohort study. Diabetologia, 2019; 62: 611-620 | sustained remission |
| 123 | Pournaras [9] | 2011 | Effect of the definition of type II diabetes remission in the evaluation of bariatric surgery for metabolic disorders | cohort | UK, Norway | 209 | Surgery | 1.2009 report (2 definitions) 2. Buchwald H, Estok R, Fahrbach K, Banel D, Jensen MD, Pories WJ, et al. Weight and type 2 diabetes after bariatric surgery: systematic review and meta-analysis. Am J Med. 2009;122(3):248–56. | - |
| 124 | Prasad [57] | 2019 | Is age a real or perceived discriminator for bariatric surgery? A long-term analysis of bariatric surgery in the elderly | cohort | USA | 166 | Surgery | 2009 report (2 definitions) | resolution |
| 125 | Pucci [158] | 2018 | Type 2 diabetes remission 2 years post Roux-en-Y gastric bypass and sleeve gastrectomy: the role of the weight loss and comparison of DiaRem and DiaBetter scores | cohort | UK | 210 | Surgery | Rubino F, Nathan DM, Eckel RH, Schauer PR, Alberti KG, Zimmet PZ, et al. Metabolic surgery in the treatment algorithm for type 2 diabetes: a joint statement by International Diabetes Organizations. Diabetes Care. 2016; 39(6):861–877. | - |
| 126 | Purnell [159] | 2016 | Type 2 diabetes remission rates after laparoscopic gastric bypass and gastric banding: Results of the longitudinal assessment of bariatric surgery study | cohort | USA | 627 | Surgery | 2009 report (1 definition) | - |
| 127 | Ramos-Levi [38] | 2013 | Which criteria should be used to define type 2 diabetes remission after bariatric surgery? | cohort | Spain | 110 | Surgery | 1. 2009 report (2 definitions). 2. Standards of medical care in diabetes-2012. American Diabetes Association. Diabetes Care. 2012 Jan; 35 Suppl 1: S11-63. | resolution |
| 128 | Ramos-Levi [160] | 2014 | Statistical models to predict type 2 diabetes remission after bariatric surgery | cohort | Spain | 141 | Surgery | 2009 report (1 definition) |  |
| 129 | Ramos-Levi [161] | 2013 | Remission of Type 2 Diabetes Mellitus Should Not Be the Foremost Goal after Bariatric Surgery | cohort | Spain | 125 | Surgery | 2009 report (1 definition) | - |
| 130 | Ruiz-Tovar [162] | 2019 | Long-term follow-up after sleeve gastrectomy versus Roux-en-Y gastric bypass versus one-anastomosis gastric bypass: a prospective randomized comparative study of weight loss and remission of comorbidities | cohort | Spain | 190 | Surgery | None | resolution |
| 131 | Samuel [163] | 2020 | Mid-term bariatric surgery outcomes for obese patients: does weight matter? | cohort | UK | 126 | Surgery | Standards of medical care in diabetes 2018. American Diabetes Association. Diabetes Care. 2018; 41 Suppl 1:S144-51 | - |
| 132 | Santoro [164] | 2012 | Sleeve Gastrectomy with Transit Bipartition: A Potent Intervention for Metabolic Syndrome and Obesity | cohort | Brazil | 281 | Surgery | None | - |
| 133 | Scally [165] | 2016 | Video Ratings of Surgical Skill and Late Outcomes of Bariatric Surgery | cohort | USA | 1041 | Surgery | None | resolution |
| 134 | Schwoerer [166] | 2017 | The effect of close postoperative follow-up on co-morbidity improvement after bariatric surgery | cohort | USA | 18629 | Surgery | None | resolution |
| 135 | Scopinaro [32] | 2017 | Prediction of Diabetes Remission at Long Term Following Biliopancreatic Diversion | cohort | Italy | 108 | Surgery | 2009 report (1 definition) | resolution |
| 136 | Seki [167] | 2017 | Five-Year-Results of Laparoscopic Sleeve Gastrectomy with Duodenojejunal Bypass for Weight Loss and Type 2 Diabetes Mellitus | cohort | Japan | 117 | Surgery | 2009 report (1 definition) | - |
| 137 | Sepulveda [168] | 2018 | Metabolic Surgery Comparing Sleeve Gastrectomy with Jejunal Bypass and Roux-en-Y Gastric Bypass in Type 2 Diabetic Patients After 3 Years | cohort | Chile | 103 | Surgery | 2009 report (1 definition) | - |
| 138 | Ser [169] | 2019 | Laparoscopic single-anastomosis duodenal-jejunal bypass with sleeve gastrectomy (SADJB-SG): Surgical risk and long-term results | cohort | Taiwan | 118 | Surgery | 2009 report (3 definitions) | resolution |
| 139 | Shah [170] | 2016 | Long-term effects of laparoscopic Roux-en-Y gastric bypass on metabolic syndrome in patients with morbid obesity | cohort | Norway | 268 | Surgery | 2009 report (2 definitions) | - |
| 140 | Shen [171] | 2019 | Validating Risk Prediction Models of Diabetes Remission After Sleeve Gastrectomy | cohort | Taiwan | 128 | Surgery | 2009 report (2 definitions) | resolution |
| 141 | Shi [172] |  | Effect of exenatide after short-time intensive insulin therapy on glycaemic remission maintenance in type 2 diabetes patients: a randomized controlled trial | RCT | China | 129 | Pharmacological | None | - |
| 142 | Sjoholm [173] | 2015 | Incidence and remission of type 2 diabetes in relation to degree of obesity at baseline and 2 year weight change: the Swedish Obese Subjects (SOS) study | cohort | Sweden | 511 | Surgery | Sjöstrom L, Peltonen M, Jacobson P, Ahlin S, Andersson-Assarsson J, Anvedenet A, et al. Association of bariatric surgery with long-term remission of type 2 diabetes and with microvascular and macrovascular complications. JAMA. 2014; 311: 2297–2304. | - |
| 143 | Sjostrom [174] | 2014 | Association of bariatric surgery with long-term remission of type 2 diabetes and with microvascular and macrovascular complications | cohort | Sweden | 603 | Surgery | 1. Definition, diagnosis, and classification of diabetes mellitus and its complications [report No. 99.2]. World Health Organization. <http://whqlibdoc.who.int/hq/1999/> who_ncd_ncs_99.2.pdf.  2. American Diabetes Association. Executive summary: standards of medical care in diabetes: 2011. Diabetes Care.2011; 34 (suppl 1):S4-S10. | - |
| 144 | Souteiro [175] | 2019 | Long-term diabetes outcomes after bariatric surgery—managing medication withdrawal | cohort | Portugal | 110 | Surgery | Brethauer SA, Kim J, el Chaar M, Papasavas P, Eisenberg D, Rogers A et al. Standardized outcomes reporting in metabolic and bariatric surgery. Surg Obes Relat Dis. 2015;11(3):489–506. | resolution |
| 145 | Souteiro [176] | 2017 | Preoperative Beta Cell Function Is Predictive of Diabetes Remission After Bariatric Surgery | cohort | Portugal | 286 | Surgery | 2009 report (1 definition) | - |
| 146 | Still [177] | 2019 | DiaRem2: Incorporating duration of diabetes to improve prediction of diabetes remission after metabolic surgery | cohort | USA | 307 | Surgery | 2009 report (2 definitions) | - |
| 147 | Still [58] | 2014 | Preoperative prediction of type 2 diabetes remission after Roux-en-Y gastric bypass surgery: A retrospective cohort study | cohort | USA | 690 | Surgery | 2009 report (2 definitions) | resolution |
| 148 | Sudan [178] | 2018 | Tailoring Bariatric Surgery: Sleeve gastrectomy, RYGB and Biliopancreatic diversion with duodenal switch | cohort | USA | 23222 | Surgery | None | resolution |
| 149 | Sudan [179] | 2017 | Comparative effectiveness of primary bariatric operations in the United States | cohort | USA | 35 841 | Surgery | None | resolution |
| 150 | Sundbom [180] | 2017 | Substantial decrease in comorbidity 5 years after gastric bypass: A population-based study from the Scandinavian obesity surgery registry | cohort | Sweden | 4056 | Surgery | None | - |
| 151 | Taha [181] | 2017 | Outcomes of One Anastomosis Gastric Bypass in 472 Diabetic Patients | cohort | Egypt | 472 | Surgery | American Diabetes Association. Standards of medical care in diabetes 2012. Diabetes Care. 2012;35(Suppl 1):S11–63 | resolution |
| 152 | Taylor [182] | 2017 | Effects of statin therapy on weight loss and diabetes in bariatric patients | cohort | USA | 557 | Pharmacological | None | resolves |
| 153 | Techagumpuch [60] | 2019 | A Prospective Randomized Control Trial: Two Years Outcome in Diabetes Control after Bariatric Surgery Comparison between Laparoscopic Sleeve Gastrectomy and Laparoscopic Roux-En-Y Gastric Bypass | RCT | Thailand | 104 | Surgery | None | - |
| 154 | Tharakan [183] | 2017 | Limitations of the DiaRem Score in Predicting Remission of Diabetes Following Roux-En-Y Gastric Bypass (RYGB) in an ethnically Diverse Population from a Single Institution in the UK | cohort | UK | 262 | Surgery | 2009 report (2 definitions) | - |
| 155 | Thereaux [184] | 2015 | Comparison of results after one year between sleeve gastrectomy and gastric bypass in patients with BMI >= 50 kg/m2 | cohort | France | 138 | Surgery | 2009 report (1 definition) | resolution |
| 156 | Toh [185] | 2018 | Five-year long-term clinical outcome after bariatric metabolic surgery: A multi-ethnic Asian population in Singapore | cohort | Singapore | 189 | Surgery | None | - |
| 157 | Valencia [59] | 2019 | The Impact of Ethnicity on Metabolic Outcomes after Bariatric Surgery | cohort | USA | 650 | Surgery | None | resolution |
| 158 | Van de Laar [186] | 2016 | Relationships between type 2 diabetes remission after gastric bypass and different weight loss metrics: Arguments against excess weight loss in metabolic surgery | cohort | Netherlands | 449 | Surgery | None | - |
| 159 | Van der Merwe [24] | 2015 | Baseline patient profiling and three-year outcome data after metabolic surgery at a South African centre of excellence | cohort | S Africa | 251 | Surgery | None | resolution |
| 160 | Velazquez-Fernandez [187] | 2019 | Development of an Interactive Outcome Estimation Tool for Laparoscopic Roux-en-Y Gastric Bypass in Mexico Based on a Cohort of 1002 Patients | cohort | Mexico | 1002 | Surgery | None | resolution |
| 161 | Victorzon [188] | 2012 | Perioperative morbidity, mortality and early outcome of the first 360 gastric bypass operations performed in a district hospital | cohort | Finland | 170 | Surgery | None | resolution |
| 162 | Viscido [189] | 2019 | Obese Patients with Type 2 Diabetes: Outcomes After Laparoscopic Sleeve Gastrectomy | cohort | Argentina | 166 | Surgery | Brethauer SA, Kim J, el Chaar M, Papasavas P, Eisenberg D, Rogers A et al. Standardized outcomes reporting in metabolic and bariatric surgery. Surg Obes Relat Dis. 2015;11(3):489–506. | resolution |
| 163 | Wadden [190] | 2019 | End-of-Trial Health Outcomes in Look AHEAD Participants who Elected to have Bariatric Surgery | cohort | USA | 196 | Surgery | None | - |
| 164 | Walker [191] | 2019 | Bariatric Surgery Among Medicare Subgroups: Short-and Long-Term Outcomes | cohort | USA | 637 | Surgery | None | - |
| 165 | Wazir [192] | 2019 | Two Years Remission of Type 2 Diabetes Mellitus after Bariatric Surgery | cohort | UK | 121 | Surgery | 1. World Health Organization. Definition and diagnosis of diabetes mellitus and intermediate hyperglycemia. Geneva: World Health Organization; 2006. http://www.who.int.  2. Report of a World Health Organization Consultation. Use of glycated haemoglobin (HbA1c) in the diagnosis of diabetes mellitus. Diabetes Res Clin Pract 2011; 93:299-309. | - |
| 166 | Wei [193] | 2018 | Metabolic surgery ameliorates cardiovascular risk in obese diabetic patients: Influence of different surgical procedures | cohort | Taiwan | 392 | Surgery | 2009 report (3 definitions) | - |
| 167 | Wood GC [194] | 2015 | Preoperative use of incretins is associated with increased diabetes remission after RYGB surgery among patients taking insulin: a retrospective cohort analysis | cohort | USA | 148 | Surgery | 2009 report (2 definitions) | resolution |
| 168 | Wood MH [195] | 2019 | Association of Race With Bariatric Surgery Outcomes | cohort | USA | 6022 | Surgery | None | - |
| 169 | Xu [196] | 2017 | Baseline red blood cell distribution width predicts long-term glycemic remission in patients with type 2 diabetes | cohort | China | 185 | Pharmacological | None | - |
| 170 | Yan [197] | 2019 | Analysis of Predictors of Type 2 Diabetes Mellitus Remission After Roux-en-Y Gastric Bypass in 101 Chinese Patients | cohort | China | 101 | Surgery | Chinese diabetes society. China guideline for type 2 diabetes. 2014. | - |
| 171 | Young [198] | 2019 | Long-term impact of bariatric surgery in diabetic nephropathy | cohort | USA | 101 | Surgery | 2009 report (1 definition) | resolution |
| 172 | Yska [30] | 2015 | Remission of type 2 diabetes mellitus in patients after different types of bariatric surgery: A population-based cohort study in the United Kingdom | cohort | UK | 2450 | Surgery | None | resolution |
| 173 | Zaman [199] | 2017 | The effects of optimal perioperative glucose control on morbidly obese patients undergoing bariatric surgery | cohort | USA | 155 | Surgery | None |  |
| 174 | Zaveri [200] | 2018 | Mid-term 4-Year Outcomes with Single Anastomosis Duodenal-Ileal Bypass with Sleeve Gastrectomy Surgery at a Single US Center | cohort | USA | 156 | Surgery | None | resolution |
| 175 | Zenti [201] | 2015 | Clinical factors that predict remission of diabetes after different bariatric surgical procedures: interdisciplinary group of bariatric surgery of Verona (G.I.C.O.V.) | cohort | Italy | 105 | Surgery | 2009 report (1 definition) | resolution |
| 176 | Zhang [202] | 2015 | The Short-Term Remission of Diabetic Nephropathy After Roux-en-Y Gastric Bypass in Chinese Patients of T2DM with Obesity | cohort | China | 101 | Surgery | 2009 report (1 definition) | - |
| 177 | Zhang [203] | 2017 | Effect of Roux-en-Y Gastric Bypass on Remission of T2D: Medium-Term Follow-up in Chinese Patients with Different BMI Obesity Class | cohort | China | 120 | Surgery | Brethauer SA, Kim J, el Chaar M, Papasavas P, Eisenberg D, Rogers A et al. Standardized outcomes reporting in metabolic and bariatric surgery. Surg Obes Relat Dis. 2015;11(3):489–506. | resolution |
| 178 | Zhu [204] | 2015 | Clinical Course of Diabetes After Gastrectomy According to Type of Reconstruction in Patients with Concurrent Gastric Cancer and Type 2 Diabetes | cohort | China | 292 | Surgery | 2009 report (1 definition) | resolution |

a Guidelines or references were not always applied to all definitions

**References**

1. International Diabetes Federation. IDF Diabetes Atlas. 8th ed. Brussels, Belgium: International Diabetes Federation. 2017 [cited 2020 Jul 30]. Available from: http://www.diabetesatlas.org.

2. Butland B, Jebb S, Kopelman P, McPherson K, Thomas S, Mardell J, et al. FORESIGHT Tackling Obesities: Future Choices — Project Report. 2nd ed. 2007 [cited 2020 Jul 31]. Available from: https://assets.publishing.service.gov.uk/government/uploads/system/uploads/attachment_data/file/287937/07-1184x-tackling-obesities-future-choices-report.pdf.

3. Commission for Healthcare Audit and Inspection. Managing diabetes: improving services for people with diabetes. 2007 [cited 2020 Jul 30]. Available from: http://www.yearofcare.co.uk/sites/default/files/pdfs/Managing_diabetes.pdf.

4. Bommer C, Sagalova V, Heesemann E, Manne-Goehler J, Atun R, Barnighausen T et al. Global economic burden of diabetes in adults: projections from 2015 to 2030. Diabetes Care. 2018;41(5):963–970. doi: 10.2337/dc17-1962

5. World Health Organization. Global Report on Diabetes 2016. 2016 [cited 2020 Jul 30]. Available from: http://apps.who.int/iris/bitstream/10665/204871/1/9789241565257_eng.pdf

6. Pories WJ, MacDonald KG Jr, Morgan EJ, Sinha MK, Dohm GL, Swanson MS et al. Surgical treatment of obesity and its effect on diabetes: 10-y follow-up. Am J Clin Nutr. 1992;55(2 Suppl):582S–585S. doi: 10.1093/ajcn/55.2.582s

7. Buchwald H, Avidor Y, Braunwald E, Jensen MD, Pories W, Fahrbach K et al. Bariatric surgery: a systematic review and meta-analysis. JAMA. 2004;292(14):1724–1737. doi: 10.1001/jama.292.14.1724

8. Buchwald H, Estok R, Fahrbach K, Banel D, Jensen MD, Pories WJ et al. Weight and type 2 diabetes after bariatric surgery: systematic review and meta-analysis. Am J Med. 2009;122(3):248–256.e5. doi: 10.1016/j.amjmed.2008.09.041

9. Pournaras DJ, Aasheim ET, Sovik TT, Andrews R, Mahon D, Welbourn R et al. Effect of the definition of type II diabetes remission in the evaluation of bariatric surgery for metabolic disorders. Br J Surg. 2012;99(1):100–103. doi: 10.1002/bjs.7704

10. Buse JB, Caprio S, Cefalu WT, Ceriello A, Del Prato S, Inzucchi SE et al. How do we define cure of diabetes? Diabetes Care. 2009;32(11):2133–2135. doi: 10.2337/dc09-9036

11. American Diabetes Association. Introduction: Standards of Medical Care in Diabetes-2019. Diabetes Care. 2019;42(Suppl 1):S1–S2. doi: 10.2337/dc19-Sint01

12. American Diabetes Association. 2. Classification and Diagnosis of Diabetes: Standards of Medical Care in Diabetes-2019. Diabetes Care. 2019;42(Suppl 1):S13–S28. doi: 10.2337/dc19-S002.

13. Lean ME, Leslie WS, Barnes AC, Brosnahan N, Thom G, McCombie L et al. Primary care-led weight management for remission of type 2 diabetes (DiRECT): an open-label, cluster-randomised trial. Lancet. 2018;391(10120):541–551. doi: 10.1016/S0140-6736(17)33102-1

14. Gregg EW, Chen H, Wagenknecht LE, Clark JM, Delahanty LM, Bantle J et al. Association of an intensive lifestyle intervention with remission of type 2 diabetes. JAMA. 2012;308(23):2489–2496. doi: 10.1001/jama.2012.67929

15. Finer S, Robb P, Cowan K, Daly A, Robertson E, Farmer A. Top ten research priorities for type 2 diabetes: results from the Diabetes UK-James Lind Alliance Priority Setting Partnership. Lancet Diabetes Endocrinol. 2017;5(12):935–936. doi: 10.1016/S2213-8587(17)30324-8

16. Diabetes UK. Diabetes UK interim position statement on remission in adults with Type 2 diabetes. 2018 [cited 2020 Jul 28]. Available from: https://www.diabetes.org.uk/resources-s3/2017-12/1302_Remission Position Statement_v1_92kb.pdf.

17. Nagi D, Hambling C, Taylor R. Remission of type 2 diabetes: a position statement from the Association of British Clinical Diabetologists (ABCD) and the Primary Care Diabetes Society (PCDS). Brit J Diab. 2019;19(1):73–76. doi: 10.15277/bjd.2019.221.

18. International Diabetes Federation. Recommendations For Managing Type 2 Diabetes In Primary Care. 2017 [cited 2020 Aug 28]. Available from: https://www.idf.org/e-library/guidelines/128-idf-clinical-practice-recommendations-for-managing-type-2-diabetes-in-primary-care.html.

19. Tricco AC, Lillie E, Zarin W, O'Brien KK, Colquhoun H, Levac D et al. PRISMA Extension for Scoping Reviews (PRISMA-ScR): Checklist and Explanation. Ann Intern Med. 2018;169(7):467–473. doi: 10.7326/M18-0850

20. Moher D, Liberati A, Tetzlaff J, Altman DG, Prisma Group. Preferred reporting items for systematic reviews and meta-analyses: the PRISMA statement. PLoS Med. 2009;6(7):e1000097. doi: 10.1371/journal.pmed.1000097

21. Karter AJ, Nundy S, Parker MM, Moffet HH, Huang ES. Incidence of remission in adults with type 2 diabetes: the diabetes & aging study. Diabetes Care. 2014;37(12):3188–3195. doi: 10.2337/dc14-0874

22. Brethauer SA, Kim J, El Chaar M, Papasavas P, Eisenberg D, Rogers A et al. Standardized outcomes reporting in metabolic and bariatric surgery. Obes Surg. 2015;25(4):587–606. doi: 10.1007/s11695-015-1645-3

23. Madsen LR, Baggesen LM, Richelsen B, Thomsen RW. Effect of Roux-en-Y gastric bypass surgery on diabetes remission and complications in individuals with type 2 diabetes: a Danish population-based matched cohort study. Diabetologia. 2019;62(4):611–620. doi: 10.1007/s00125-019-4816-2

24. van der Merwe M-T, Fetter G, Naidoo S, Wilson R, Drabble N, Gonçalves D et al. Baseline patient profiling and three-year outcome data after metabolic surgery at a South African centre of excellence. J Endocrinol Metab Diabetes S Afr. 2015;20(3):115–126. doi: 10.1080/16089677.2015.1085700

25. Bhasker AG, Remedios C, Batra P, Sood A, Shaikh S, Lakdawala M. Predictors of Remission of T2DM and Metabolic Effects after Laparoscopic Roux-en-y Gastric Bypass in Obese Indian Diabetics-a 5-Year Study. Obes Surg. 2015;25(7):1191–1197. doi: 10.1007/s11695-014-1501-x

26. Dicker D, Yahalom R, Comaneshter DS, Vinker S. Long-Term Outcomes of Three Types of Bariatric Surgery on Obesity and Type 2 Diabetes Control and Remission. Obes Surg. 2016;26(8):1814–1820. doi: 10.1007/s11695-015-2025-8

27. Kular KS, Manchanda N, Cheema GK. Seven Years of Mini-Gastric Bypass in Type II Diabetes Patients with a Body Mass Index <35 kg/m(2). Obes Surg. 2016;26(7):1457–1462. doi: 10.1007/s11695-015-1941-y

28. Moh MC, Cheng A, Tan CH, Lim BK, Tan BC, Ng D et al. Metabolic Surgery Diabetes Remission (MDR) Score: a New Preoperative Scoring System for Predicting Type 2 Diabetes Remission at 1 Year After Metabolic Surgery in the Singapore Multi-ethnic Asian Setting. Obes Surg. 2020. doi: 10.1007/s11695-020-04576-3

29. Friedman AN, Wang J, Wahed AS, Docherty NG, Fennern E, Pomp A et al. The Association Between Kidney Disease and Diabetes Remission in Bariatric Surgery Patients With Type 2 Diabetes. Am J Kidney Dis. 2019;74(6):761–770. doi: 10.1053/j.ajkd.2019.05.013

30. Yska JP, van Roon EN, de Boer A, Leufkens HG, Wilffert B, de Heide LJ et al. Remission of Type 2 Diabetes Mellitus in Patients After Different Types of Bariatric Surgery: A Population-Based Cohort Study in the United Kingdom. JAMA Surg. 2015;150(12):1126–1133. doi: 10.1001/jamasurg.2015.2398

31. Nora M, Morais T, Almeida R, Guimaraes M, Monteiro MP. Should Roux-en-Y gastric bypass biliopancreatic limb length be tailored to achieve improved diabetes outcomes? Medicine. 2017;96(48):e8859. doi: 10.1097/MD.0000000000008859

32. Scopinaro N, Adami GF, Bruzzi P, Cordera R. Prediction of Diabetes Remission at Long Term Following Biliopancreatic Diversion. Obes Surg. 2017;27(7):1705–1708. doi: 10.1007/s11695-017-2555-3

33. Douglas IJ, Bhaskaran K, Batterham RL, Smeeth L. Bariatric Surgery in the United Kingdom: A Cohort Study of Weight Loss and Clinical Outcomes in Routine Clinical Care. PLoS Med. 2015;12(12):e1001925. doi: 10.1371/journal.pmed.1001925

34. Arterburn D, Bogart A, Coleman KJ, Haneuse S, Selby JV, Sherwood NE et al. Comparative effectiveness of bariatric surgery vs. nonsurgical treatment of type 2 diabetes among severely obese adults. Obes Res Clin Pract. 2013;7(4):e258–e268. doi: 10.1016/j.orcp.2012.08.196

35. Arterburn DE, Bogart A, Sherwood NE, Sidney S, Coleman KJ, Haneuse S et al. A multisite study of long-term remission and relapse of type 2 diabetes mellitus following gastric bypass. Obes Surg. 2013;23(1):93–102.

36. Bohula EA, Scirica BM, Inzucchi SE, McGuire DK, Keech AC, Smith SR et al. Effect of lorcaserin on prevention and remission of type 2 diabetes in overweight and obese patients (CAMELLIA-TIMI 61): a randomised, placebo-controlled trial. Lancet. 2018;392(10161):2269–2279. doi: 10.1016/S0140-6736(18)32328-6

37. McTigue KM, Wellman R, Nauman E, Anau J, Coley RY, Odor A et al. Comparing the 5-Year Diabetes Outcomes of Sleeve Gastrectomy and Gastric Bypass: The National Patient-Centered Clinical Research Network (PCORNet) Bariatric Study. JAMA Surg. 2020;155(5):e200087. doi: 10.1001/jamasurg.2020.0087

38. Ramos-Levi AM, Cabrerizo L, Matia P, Sanchez-Pernaute A, Torres AJ, Rubio MA. Which criteria should be used to define type 2 diabetes remission after bariatric surgery? BMC Surg. 2013;13:8.

39. Alhambra-Exposito MR, Molina-Puerta MJ, Prior-Sanchez MI, Manzano-Garcia G, Calanas-Continente A, Galvez-Moreno MA. Variations in diabetes remission rates after bariatric surgery in Spanish adults according to the use of different diagnostic criteria for diabetes. BMC Endocr Disord. 2017;17(1):51. doi: 10.1186/s12902-017-0201-7

40. Pereyra-Garcia Castro FM, Oliva Garcia JG, Garcia Nunez MA, Garcia Bray BF, Suarez Llanos JP, Moneva Arce ME et al. Efficacy in type 2 diabetes mellitus remission in patients undergoing bariatric surgery. Endocrinol Diabetes Nutr. 2019;66(1):56–61. doi: 10.1016/j.endinu.2018.08.007

41. Blackstone R, Bunt JC, Cortes MC, Sugerman HJ. Type 2 diabetes after gastric bypass: remission in five models using HbA1c, fasting blood glucose, and medication status. Surg Obes Relat Dis. 2012;8(5):548–555. doi: 10.1016/j.soard.2012.05.005

42. Murphy R, Clarke MG, Evennett NJ, John Robinson S, Lee Humphreys M, Hammodat H et al. Laparoscopic Sleeve Gastrectomy Versus Banded Roux-en-Y Gastric Bypass for Diabetes and Obesity: a Prospective Randomised Double-Blind Trial. Obes Surg. 2018;28(2):293–302. doi: 10.1007/s11695-017-2872-6

43. Panunzi S, De Gaetano A, Carnicelli A, Mingrone G. Predictors of remission of diabetes mellitus in severely obese individuals undergoing bariatric surgery: do BMI or procedure choice matter? A meta-analysis. Ann Surg. 2015;261(3):459–67. doi: 10.1097/SLA.0000000000000863. PubMed PMID: 25361217.

44. Gloy VL, Briel M, Bhatt DL, Kashyap SR, Schauer PR, Mingrone G et al. Bariatric surgery versus non-surgical treatment for obesity: a systematic review and meta-analysis of randomised controlled trials. BMJ. 2013;347:f5934. doi: 10.1136/bmj.f5934

45. Muller-Stich BP, Senft JD, Warschkow R, Kenngott HG, Billeter AT, Vit G et al. Surgical versus medical treatment of type 2 diabetes mellitus in nonseverely obese patients: a systematic review and meta-analysis. Ann Surg. 2015;261(3):421–429. doi: 10.1097/SLA.0000000000001014

46. Rao WS, Shan CX, Zhang W, Jiang DZ, Qiu M. A meta-analysis of short-term outcomes of patients with type 2 diabetes mellitus and BMI </= 35 kg/m2 undergoing Roux-en-Y gastric bypass. World J Surg. 2015;39(1):223–230. doi: 10.1007/s00268-014-2751-4

47. Lee SK, Heo Y, Park JM, Kim YJ, Kim SM, Park JM, et al. Roux-en-Y Gastric Bypass vs. Sleeve Gastrectomy vs. Gastric Banding: The First Multicenter Retrospective Comparative Cohort Study in Obese Korean Patients. Yonsei Med J. 2016;57(4):956–962. doi: 10.3349/ymj.2016.57.4.956

48. Ahuja A, Tantia O, Chaudhuri T, Khanna S, Seetharamaiah S, Majumdar K et al. Predicting remission of diabetes post metabolic surgery: a comparison of ABCD, diarem, and DRS scores. Obes Surg. 2018;28(7):2025–2031. doi: 10.1007/s11695-018-3136-9

49. Aminian A, Daigle CR, Romero-Talamas H, Kashyap SR, Kirwan JP, Brethauer SA et al. Risk prediction of complications of metabolic syndrome before and 6 years after gastric bypass. Surg Obes Relat Dis. 2014;10(4):576–582. doi: 10.1016/j.soard.2014.01.025

50. Araia M, Wood M, Kroll J, Abou-Samra A, Seyoum B. Resolution of diabetes after bariatric surgery among predominantly African-American patients: race has no effect in remission of diabetes after bariatric surgery. Obes Surg. 2014;24(6):835–840. doi: 10.1007/s11695-014-1187-0

51. Aron-Wisnewsky J, Sokolovska N, Liu Y, Comaneshter DS, Vinker S, Pecht T et al. The advanced-DiaRem score improves prediction of diabetes remission 1 year post-Roux-en-Y gastric bypass. Diabetologia. 2017;60(10):1892–1902. doi: 10.1007/s00125-017-4371-7

52. Boza C, Valderas P, Daroch DA, Leon FI, Salinas JP, Barros DA et al. Metabolic surgery: roux-en-Y gastric bypass and variables associated with diabetes remission in patients with BMI <35. Obes Surg. 2014;24(8):1391–1397. doi: 10.1007/s11695-014-1218-x

53. Chen JC, Hsu NY, Lee WJ, Chen SC, Ser KH, Lee YC. Prediction of type 2 diabetes remission after metabolic surgery: a comparison of the individualized metabolic surgery score and the ABCD score. Surg Obes Relat Dis. 2018;14(5):640–645.

54. Craig Wood G, Horwitz D, Still CD, Mirshahi T, Benotti P, Parikh M et al. Performance of the DiaRem Score for Predicting Diabetes Remission in Two Health Systems Following Bariatric Surgery Procedures in Hispanic and non-Hispanic White Patients. Obes Surg. 2018;28(1):61–68. doi: 10.1007/s11695-017-2799-y

55. Hayes S, Napolitano MA, Lent MR, Wood GC, Gerhard GS, Irving BA et al. The effect of insurance status on pre- and post-operative bariatric surgery outcomes. Obes Surg. 2015;25(1):191–194. doi: 10.1007/s11695-014-1478-5

56. Lee WJ, Chong K, Ser KH, Chen JC, Lee YC, Chen SC et al. C-peptide predicts the remission of type 2 diabetes after bariatric surgery. Obes Surg. 2012;22(2):293–298. doi: 10.1007/s11695-011-0565-0

57. Prasad J, Vogels E, Dove JT, Wood C, Petrick AT, Parker DM. Is age a real or perceived discriminator for bariatric surgery? A long-term analysis of bariatric surgery in the elderly. Surg Obes Relat Dis. 2019;15(5):725–731. doi: 10.1016/j.soard.2018.12.019

58. Still CD, Wood GC, Benotti P, Petrick AT, Gabrielsen J, Strodel WE et al. Preoperative prediction of type 2 diabetes remission after Roux-en-Y gastric bypass surgery: a retrospective cohort study. Lancet Diabetes Endocrinol. 2014;2(1):38–45. doi: 10.1016/S2213-8587(13)70070-6

59. Valencia A, Garcia LC, Morton J. The Impact of Ethnicity on Metabolic Outcomes After Bariatric Surgery. J Surg Res. 2019;236:345–351. doi: 10.1016/j.jss.2018.09.061

60. Techagumpuch A, Thanavachirsin K, Udomsawaengsup S. A prospective randomized control trial: two years outcome in diabetes control after bariatric surgery comparison between laparoscopic sleeve gastrectomy and laparoscopic roux-en-Y gastric bypass. J Med Assoc Thai. 2019;102(3):298–303.

61. Vickers AJ, Basch E, Kattan MW. Against diagnosis. Ann Intern Med. 2008;149(3):200–203. doi: 10.7326/0003-4819-149-3-200808050-00010

62. World Health Organization, International Diabetes Federation. Definition and diagnosis of diabetes mellitus and intermediate hyperglycaemia : report of a WHO/IDF consultation Geneva: World Health Organization. 2006 [cited 2020 Jul 25]. Available from: https://apps.who.int/iris/handle/10665/43588.

63. Kveim Lie A, Greene JA. From Ariadne's Thread to the Labyrinth Itself—Nosology and the Infrastructure of Modern Medicine. N Engl J Med. 2020;382(13):1273–1277. doi: 10.1056/NEJMms1913140

64. World Health Organization. Use of Glycated Haemoglobin (HbA1c) in the Diagnosis of Diabetes Mellitus. Abbreviated Report of a WHO Consultation. 2011 [cited 2020 Jul 30]. Available from: https://www.who.int/diabetes/publications/report-hba1c_2011.pdf?ua=1.

65. Abd Ellatif ME, Abdallah E, Askar W, Thabet W, Aboushady M, Abbas AE et al. Long term predictors of success after laparoscopic sleeve gastrectomy. Int J Surg. 2014;12(5):504–508. doi: 10.1016/j.ijsu.2014.02.008

66. Abu-Abeid A, Lessing Y, Pencovich N, Dayan D, Klausner JM, Abu-Abeid S. Diabetes resolution after one anastomosis gastric bypass. Surg Obes Relat Dis. 2018;14(2):181–185. doi: 10.1016/j.soard.2017.10.023

67. Adams TD, Davidson LE, Litwin SE, Kolotkin RL, LaMonte MJ, Pendleton RC et al. Health benefits of gastric bypass surgery after 6 years. JAMA. 2012;308(11):1122–1131. doi: 10.1001/2012.jama.11164

68. Al-Khyatt W, Bull CA, Awad S, Ahmed J. Laparoscopic Roux en-Y Gastric Bypass Using a Modified Retrocolic-Supracolic Approach: Outcomes from 300 Patients. World J Surg. 2016;40(8):1918–1924. doi: 10.1007/s00268-016-3478-1

69. Almalki OM, Lee WJ, Chong K, Ser KH, Lee YC, Chen SC. Laparoscopic gastric bypass for the treatment of type 2 diabetes: a comparison of Roux-en-Y versus single anastomosis gastric bypass. Surg Obes Relat Dis. 2018;14(4):509–515. doi: 10.1016/j.soard.2017.12.022

70. Al-Sabah S, Almazeedi S, Alosaimi S, Al-Mulla A, Ali DAM, Al-Elewah A et al. Remission of Type 2 diabetes mellitus after laparoscopic sleeve gastrectomy. World Journal of Laparoscopic Surgery. 2014;7(3):121–124.

71. Aminian A, Vidal J, Salminen P, Still CD, Nor Hanipah Z, Sharma G et al. Late Relapse of Diabetes After Bariatric Surgery: Not Rare, but Not a Failure. Diabetes Care. 2020;43(3):534–540. doi: 10.2337/dc19-1057

72. Aminian A, Brethauer SA, Andalib A, Nowacki AS, Jimenez A, Corcelles R et al. Individualized Metabolic Surgery Score: Procedure Selection Based on Diabetes Severity. Ann Surg. 2017;266(4):650–657. doi: 10.1097/SLA.0000000000002407

73. Aminian A, Brethauer SA, Andalib A, Punchai S, Mackey J, Rodriguez J et al. Can Sleeve Gastrectomy “Cure” Diabetes? Long-term Metabolic Effects of Sleeve Gastrectomy in Patients With Type 2 Diabetes. Ann Surg. 2016;264(4):674–681. doi: 10.1097/SLA.0000000000001857

74. Ardestani A, Rhoads D, Tavakkoli A. Insulin cessation and diabetes remission after bariatric surgery in adults with insulin-treated type 2 diabetes. Diabetes Care. 2015;38(4):659–664. doi: 10.2337/dc14-1751

75. Aung L, Lee WJ, Chen SC, Ser KH, Wu CC, Chong K et al. Bariatric Surgery for Patients With Early-Onset vs Late-Onset Type 2 Diabetes. JAMA Surg. 2016;151(9):798–805. doi: 10.1001/jamasurg.2016.1130

76. Bayham BE, Greenway FL, Bellanger DE, O'Neil CE. Early resolution of type 2 diabetes seen after Roux-en-Y gastric bypass and vertical sleeve gastrectomy. Diabetes Technol Ther. 2012;14(1):30–34. doi: 10.1089/dia.2011.0151

77. Behbehani F, Ammori BJ, New JP, Summers LK, Soran H, Syed AA. Metabolic outcomes 2 years following gastric bypass surgery in people with type 2 diabetes: an observational cohort study. QJM. 2014;107(9):721–726. doi: 10.1093/qjmed/hcu060

78. Bhasker AG, Dixon JB, Lakdawala M. Selection of Bypass vs Sleeve for the Management of Type-2 Diabetes in Severely Obese: Could Ethnicity Play a Role? Obes Surg. 2018;28(10):3073–3079. doi: 10.1007/s11695-018-3294-9

79. Biertho L, Lebel S, Marceau S, Hould FS, Lescelleur O, Marceau P et al. Laparoscopic sleeve gastrectomy: with or without duodenal switch? A consecutive series of 800 cases. Dig Surg. 2014;31(1):48–54. doi: 10.1159/000354313

80. Brethauer SA, Aminian A, Romero-Talamas H, Batayyah E, Mackey J, Kennedy L et al. Can diabetes be surgically cured? Long-term metabolic effects of bariatric surgery in obese patients with type 2 diabetes mellitus. Ann Surg. 2013;258(4):628–636.

81. Bruno G, Gruden G, Barutta F, Cavallo Perin P, Morino M, Toppino M. What is the impact of sleeve gastrectomy and gastric bypass on metabolic control of diabetes? A clinic-based cohort of Mediterranean diabetic patients. Surg Obes Relat Dis. 2015;11(5):1014–1019.

82. Camerini GB, Papadia FS, Carlini F, Catalano M, Adami GF, Scopinaro N. The long-term impact of biliopancreatic diversion on glycemic control in the severely obese with type 2 diabetes mellitus in relation to preoperative duration of diabetes. Surg Obes Relat Dis. 2016;12(2):345–349. doi: 10.1016/j.soard.2015.05.012

83. Chen A, Huang Z, Wan X, Deng W, Wu J, Li L et al. Attitudes toward diabetes affect maintenance of drug-free remission in patients with newly diagnosed type 2 diabetes after short-term continuous subcutaneous insulin infusion treatment. Diabetes Care. 2012;35(3):474–481. doi: 10.2337/dc11-1638

84. Chen Y, Corsino L, Shantavasinkul PC, Grant J, Portenier D, Ding L et al. Gastric Bypass Surgery Leads to Long-term Remission or Improvement of Type 2 Diabetes and Significant Decrease of Microvascular and Macrovascular Complications. Ann Surg. 2016;263(6):1138–1142. doi: 10.1097/SLA.0000000000001509

85. Chikunguwo SM, Wolfe LG, Dodson P, Meador JG, Baugh N, Clore JN et al. Analysis of factors associated with durable remission of diabetes after Roux-en-Y gastric bypass. Surg Obes Relat Dis. 2010;6(3):254–259. doi: 10.1016/j.soard.2009.11.003

86. Cottam A, Cottam D, Zaveri H, Cottam S, Surve A, Medlin W et al. An Analysis of Mid-Term Complications, Weight Loss, and Type 2 Diabetes Resolution of Stomach Intestinal Pylorus-Sparing Surgery (SIPS) Versus Roux-En-Y Gastric Bypass (RYGB) with Three-Year Follow-Up. Obes Surg. 2018;28(9):2894–2902. doi: 10.1007/s11695-018-3309-6

87. Courcoulas AP, King WC, Belle SH, Berk P, Flum DR, Garcia L et al. Seven-Year Weight Trajectories and Health Outcomes in the Longitudinal Assessment of Bariatric Surgery (LABS) Study. JAMA Surg. 2018;153(5):427–434. doi: 10.1001/jamasurg.2017.5025

88. Dambha-Miller H, Day AJ, Strelitz J, Irving G, Griffin SJ. Behaviour change, weight loss and remission of Type 2 diabetes: a community-based prospective cohort study. Diabet Med. 2020;37(4):681–688. doi: 10.1111/dme.14122

89. Dang JT, Sheppard C, Kim D, Switzer N, Shi X, Tian C et al. Predictive factors for diabetes remission after bariatric surgery. Can J Surg. 2019;62(5):315–319. doi: 10.1503/cjs.014516

90. Davies SW, Efird JT, Guidry CA, Penn RI, Sawyer RG, Schirmer BD et al. Long-term diabetic response to gastric bypass. J Surg Res. 2014;190(2):498–503. doi: 10.1016/j.jss.2014.01.047

91. Debedat J, Sokolovska N, Coupaye M, Panunzi S, Chakaroun R, Genser L et al. Long-term Relapse of Type 2 Diabetes After Roux-en-Y Gastric Bypass: Prediction and Clinical Relevance. Diabetes Care. 2018;41(10):2086–2095. doi: 10.2337/dc18-0567

92. de Oliveira VLP, Martins GP, Mottin CC, Rizzolli J, Friedman R. Predictors of Long-Term Remission and Relapse of Type 2 Diabetes Mellitus Following Gastric Bypass in Severely Obese Patients. Obes Surg. 2018;28(1):195–203. doi: 10.1007/s11695-017-2830-3

93. Dicker D, Golan R, Aron-Wisnewsky J, Zucker JD, Sokolowska N, Comaneshter DS et al. Prediction of Long-Term Diabetes Remission After RYGB, Sleeve Gastrectomy, and Adjustable Gastric Banding Using DiaRem and Advanced-DiaRem Scores. Obes Surg. 2019;29(3):796–804. doi: 10.1007/s11695-018-3583-3

94. Dixon JB, Chuang LM, Chong K, Chen SC, Lambert GW, Straznicky NE et al. Predicting the glycemic response to gastric bypass surgery in patients with type 2 diabetes. Diabetes Care. 2013;36(1):20–26. doi: 10.2337/dc12-0779

95. Dorman RB, Serrot FJ, Miller CJ, Slusarek BM, Sampson BK, Buchwald H et al. Case-matched outcomes in bariatric surgery for treatment of type 2 diabetes in the morbidly obese patient. Ann Surg. 2012;255(2):287–293. doi: 10.1097/SLA.0b013e318232b033

96. Du X, Fu XH, Shi L, Hu JK, Zhou ZG, Cheng Z. Effects of Laparoscopic Roux-en-Y Gastric Bypass on Chinese Type 2 Diabetes Mellitus Patients with Different Levels of Obesity: Outcomes After 3 Years' Follow-Up. Obes Surg. 2018;28(3):702–711. doi: 10.1007/s11695-017-2903-3

97. Durmush EK, Ermerak G, Durmush D. Short-term outcomes of sleeve gastrectomy for morbid obesity: Does staple line reinforcement matter? Obes Surg. 2014;24(7):1109–1116.

98. Egan RJ, Johnson AB, Morgan JDT, Norton SA. The Impact of Laparoscopic Adjustable Gastric Banding on an NHS Cohort of Type 2 Diabetics: a Prospective Cohort Study. Obes Surg. 2016;26(9):2006–2013. doi: 10.1007/s11695-015-2039-2

99. English TM, Malkani S, Kinney RL, Omer A, Dziewietin MB, Perugini R. Predicting remission of diabetes after RYGB surgery following intensive management to optimize preoperative glucose control. Obes Surg. 2015;25(1):1–6. doi: 10.1007/s11695-014-1339-2

100. Esposito K, Maiorino MI, Petrizzo M, Bellastella G, Giugliano D. The effects of a Mediterranean diet on the need for diabetes drugs and remission of newly diagnosed type 2 diabetes: follow-up of a randomized trial. Diabetes Care. 2014;37(7):1824–1830. doi: 10.2337/dc13-2899

101. Finno P, Osorio J, Garcia-Ruiz-de-Gordejuela A, Casajoana A, Sorribas M, Admella V et al. Single Versus Double-Anastomosis Duodenal Switch: Single-Site Comparative Cohort Study in 440 Consecutive Patients. Obes Surg. 2020. doi: 10.1007/s11695-020-04566-5

102. Girundi MG. Type 2 Diabetes Mellitus remission eighteen months after Roux-en-Y gastric bypass. Rev Col Bras Cir. 2016;43(3):149–153. doi: 10.1590/0100-69912016003002

103. Guerreiro V, Neves JS, Salazar D, Ferreira MJ, Oliveira SC, Souteiro P et al. Long-Term Weight Loss and Metabolic Syndrome Remission after Bariatric Surgery: The Effect of Sex, Age, Metabolic Parameters and Surgical Technique - A 4-Year Follow-Up Study. Obes Facts. 2019;12(6):639–652. doi: 10.1159/000503753

104. Gullick AA, Graham LA, Richman J, Kakade M, Stahl R, Grams J. Association of race and socioeconomic status with outcomes following laparoscopic Roux-en-Y gastric bypass. Obes Surg. 2015;25(4):705–711. doi: 10.1007/s11695-014-1447-z

105. Gulliford MC, Booth HP, Reddy M, Charlton J, Fildes A, Prevost AT et al. Effect of Contemporary Bariatric Surgical Procedures on Type 2 Diabetes Remission. A Population-Based Matched Cohort Study. Obes Surg. 2016;26(10):2308–2315. doi: 10.1007/s11695-016-2103-6

106. Hall TC, Pellen MG, Sedman PC, Jain PK. Preoperative factors predicting remission of type 2 diabetes mellitus after Roux-en-Y gastric bypass surgery for obesity. Obes Surg. 2010;20(9):1245–1250. doi: 10.1007/s11695-010-0198-8

107. Haider KS, Haider A, Saad F, Doros G, Hanefeld M, Dhindsa S et al. Remission of type 2 diabetes following long-term treatment with injectable testosterone undecanoate in patients with hypogonadism and type 2 diabetes: 11-year data from a real-world registry study. Diabetes Obes Metab. 2020;19. doi: 10.1111/dom.14122

108. Hariri K, Guevara D, Jayaram A, Kini SU, Herron DM, Fernandez-Ranvier G. Preoperative insulin therapy as a marker for type 2 diabetes remission in obese patients after bariatric surgery. Surg Obes Relat Dis. 2018;14(3):332–337. doi: 10.1016/j.soard.2017.11.016

109. Haruta H, Kasama K, Ohta M, Sasaki A, Yamamoto H, Miyazaki Y et al. Long-Term Outcomes of Bariatric and Metabolic Surgery in Japan: Results of a Multi-Institutional Survey. Obes Surg. 2017;27(3):754–762. doi: 10.1007/s11695-016-2361-3

110. Hatoum IJ, Blackstone R, Hunter TD, Francis DM, Steinbuch M, Harris JL et al. Clinical Factors Associated With Remission of Obesity-Related Comorbidities After Bariatric Surgery. JAMA Surg. 2016;151(2):130–137. doi: 10.1001/jamasurg.2015.3231

111. Hayes MT, Hunt LA, Foo J, Tychinskaya Y, Stubbs RS. A model for predicting the resolution of type 2 diabetes in severely obese subjects following Roux-en Y gastric bypass surgery. Obes Surg. 2011;21(7):910–916. doi: 10.1007/s11695-011-0370-9

112. Hoerger TJ, Zhang P, Segel JE, Kahn HS, Barker LE, Couper S. Cost-effectiveness of bariatric surgery for severely obese adults with diabetes. Diabetes Care. 2010;33(9):1933–1939. doi: 10.2337/dc10-0554

113. Hofso D, Fatima F, Borgeraas H, Birkeland KI, Gulseth HL, Hertel JK et al. Gastric bypass versus sleeve gastrectomy in patients with type 2 diabetes (Oseberg): a single-centre, triple-blind, randomised controlled trial. Lancet Diabetes Endocrinol. 2019;7(12):912–924. doi: 10.1016/S2213-8587(19)30344-4

114. Honarmand K, Chetty K, Vanniyasingam T, Anvari M, Chetty VT. Type 2 diabetes remission rates 1-year post-Roux-en-Y gastric bypass and validation of the DiaRem score: the Ontario Bariatric Network experience. Clin Obes. 2017;7(3):176–182. doi: 10.1111/cob.12189

115. Hsu CC, Almulaifi A, Chen JC, Ser KH, Chen SC, Hsu KC et al. Effect of Bariatric Surgery vs Medical Treatment on Type 2 Diabetes in Patients With Body Mass Index Lower Than 35: Five-Year Outcomes. JAMA Surg. 2015;150(12):1117–1124. doi: 10.1001/jamasurg.2015.2602

116. Hussain A, El-Hasani S. Short- and Mid-term Outcomes of 527 One Anastomosis Gastric Bypass/Mini-Gastric Bypass (OAGB/MGB) Operations: Retrospective Study. Obes Surg. 2019;29(1):262–267. doi: 10.1007/s11695-018-3516-1

117. Iacobellis G, Xu C, Campo RE, De La Cruz-Munoz NF. Predictors of short-term diabetes remission after laparoscopic Roux-en-Y gastric bypass. Obes Surg. 2015;25(5):782–787. doi: 10.1007/s11695-014-1477-6

118. Ikramuddin S, Korner J, Lee WJ, Bantle JP, Thomas AJ, Connett JE et al. Durability of Addition of Roux-en-Y Gastric Bypass to Lifestyle Intervention and Medical Management in Achieving Primary Treatment Goals for Uncontrolled Type 2 Diabetes in Mild to Moderate Obesity: A Randomized Control Trial. Diabetes Care. 2016;39(9):1510–1518. doi: 10.2337/dc15-2481

119. Inabnet WB III, Winegar DA, Sherif B, Sarr MG. Early outcomes of bariatric surgery in patients with metabolic syndrome: an analysis of the bariatric outcomes longitudinal database. J Am Coll Surg 2012;214(4):550-6; discussion 6–7. doi: 10.1016/j.jamcollsurg.2011.12.019.

120. Jakobsen GS, Smastuen MC, Sandbu R, Nordstrand N, Hofso D, Lindberg M et al. Association of Bariatric Surgery vs Medical Obesity Treatment With Long-term Medical Complications and Obesity-Related Comorbidities. JAMA. 2018;319(3):291–301. doi: 10.1001/jama.2017.21055

121. Jans A, Naslund I, Ottosson J, Szabo E, Naslund E, Stenberg E. Duration of type 2 diabetes and remission rates after bariatric surgery in Sweden 2007-2015: A registry-based cohort study. PLoS Med. 2019;16(11):e1002985. doi: 10.1371/journal.pmed.1002985. PubMed PMID: 31747392.

122. Jimenez A, Casamitjana R, Flores L, Viaplana J, Corcelles R, Lacy A et al. Long-term effects of sleeve gastrectomy and Roux-en-Y gastric bypass surgery on type 2 diabetes mellitus in morbidly obese subjects. Ann Surg. 2012;256(6):1023–1029. doi: 10.1097/SLA.0b013e318262ee6b

123. Jimenez A, Ceriello A, Casamitjana R, Flores L, Viaplana-Masclans J, Vidal J. Remission of type 2 diabetes after Roux-en-Y gastric bypass or sleeve gastrectomy is associated with a distinct glycemic profile. Ann Surg. 2015;261(2):316–322. doi: 10.1097/SLA.0000000000000586

124. Jonsson E, Ornstein P, Goine H, Hedenbro JL. Diabetes Resolution and Work Absenteeism After Gastric Bypass: a 6-Year Study. Obes Surg. 2017;27(9):2246–2252. doi: 10.1007/s11695-017-2642-5

125. Kaska L, Proczko M, Kobiela J, Stefaniak TJ, Sledzinski Z. Dynamics of type 2 diabetes mellitus laboratory remission after Roux-en-Y gastric bypass in patients with body mass index lower than 35 kg/m2 and higher than 35 kg/m2 in a 3-year observation period. Wideochirurgia I Inne Techniki Maloinwazyjne. 2014;9(4):523–530.

126. Khalaj A, Mousapour P, Motamedi MAK, Mahdavi M, Valizadeh M, Hosseinpanah F et al. Comparing the Efficacy and Safety of Roux-en-Y Gastric Bypass with One-Anastomosis Gastric Bypass with a Biliopancreatic Limb of 200 or 160 cm: 1-Year Results of the Tehran Obesity Treatment Study (TOTS). Obes Surg. 2020. doi: 10.1007/s11695-020-04681-3

127. Kim JW, Cheong JH, Hyung WJ, Choi SH, Noh SH. Outcome after gastrectomy in gastric cancer patients with type 2 diabetes. World J Gastroenterol. 2012;18(1):49-54. doi: 10.3748/wjg.v18.i1.49. PubMed Central PMCID: PMCPMC3251805.

128. Kim S, Richards WO. Long-term follow-up of the metabolic profiles in obese patients with type 2 diabetes mellitus after Roux-en-Y gastric bypass. Ann Surg. 2010;251(6):1049–1055. doi: 10.1097/SLA.0b013e3181d9769b

129. Kothari SN, Borgert AJ, Kallies KJ, Baker MT, Grover BT. Long-term (>10-year) outcomes after laparoscopic Roux-en-Y gastric bypass. Surg Obes Relat Dis. 2017;13(6):972–978. doi: 10.1016/j.soard.2016.12.011

130. Lager CJ, Esfandiari NH, Luo Y, Subauste AR, Kraftson AT, Brown MB et al. Metabolic Parameters, Weight Loss, and Comorbidities 4 Years After Roux-en-Y Gastric Bypass and Sleeve Gastrectomy. Obes Surg. 2018;28(11):3415–3423. doi: 10.1007/s11695-018-3346-1

131. Lean MEJ, Leslie WS, Barnes AC, Brosnahan N, Thom G, McCombie L et al. Durability of a primary care-led weight-management intervention for remission of type 2 diabetes: 2-year results of the DiRECT open-label, cluster-randomised trial. Lancet Diabetes Endocrinol. 2019;7(5):344–355. doi: 10.1016/S2213-8587(19)30068-3

132. Lee MH, Lee WJ, Chong K, Chen JC, Ser KH, Lee YC et al. Predictors of long-term diabetes remission after metabolic surgery. J Gastrointest Surg. 2015;19(6):1015–1021. doi: 10.1007/s11605-015-2808-1

133. Lee PC, Tham KW, Ganguly S, Tan HC, Eng AKH, Dixon JB. Ethnicity Does Not Influence Glycemic Outcomes or Diabetes Remission After Sleeve Gastrectomy or Gastric Bypass in a Multiethnic Asian Cohort. Obes Surg. 2018;28(6):1511–1518. doi: 10.1007/s11695-017-3050-6

134. Lee W, Ahn SH, Lee JH, Park DJ, Lee HJ, Kim HH et al. Comparative study of diabetes mellitus resolution according to reconstruction type after gastrectomy in gastric cancer patients with diabetes mellitus. Obes Surg. 2012;22(8):1238–1243. doi: 10.1007/s11695-011-0580-1

135. Lee WJ, Almulaifi A, Chong K, Chen SC, Tsou JJ, Ser KH et al. The Effect and Predictive Score of Gastric Bypass and Sleeve Gastrectomy on Type 2 Diabetes Mellitus Patients with BMI < 30 kg/m(2). Obes Surg. 2015;25(10):1772–1778. doi: 10.1007/s11695-015-1603-0

136. Lee WJ, Almulaifi A, Chong K, Yao WC, Tsou JJ, Ser KH et al. Bariatric versus diabetes surgery after five years of follow up. Asian J Surg. 2016;39(2):96–102. doi: 10.1016/j.asjsur.2015.04.001

137. Lee WJ, Chong K, Aung L, Chen SC, Ser KH, Lee YC. Metabolic Surgery for Diabetes Treatment: Sleeve Gastrectomy or Gastric Bypass? World J Surg. 2017;41(1):216–223. doi: 10.1007/s00268-016-3690-z

138. Lee WJ, Chong K, Chen SC, Zachariah J, Ser KH, Lee YC et al. Preoperative Prediction of Type 2 Diabetes Remission After Gastric Bypass Surgery: a Comparison of DiaRem Scores and ABCD Scores. Obes Surg. 2016;26(10):2418–2424. doi: 10.1007/s11695-016-2120-5

139. Lee WJ, Hur KY, Lakadawala M, Kasama K, Wong SK, Chen SC et al. Predicting success of metabolic surgery: age, body mass index, C-peptide, and duration score. Surg Obes Relat Dis. 2013;9(3):379–384. doi: 10.1016/j.soard.2012.07.015

140. Lemus R, Karni D, Hong D, Gmora S, Breau R, Anvari M. The impact of bariatric surgery on insulin-treated type 2 diabetes patients. Surg Endosc. 2018;32(2):990–1001. doi: 10.1007/s00464-017-5777-5

141. Liang H, Cao Q, Liu H, Guan W, Wong C, Tong D. The Predictive Factors for Diabetic Remission in Chinese Patients with BMI > 30 kg/m(2) and BMI < 30 kg/m(2) Are Different. Obes Surg. 2018;28(7):1943–1949. doi: 10.1007/s11695-017-3106-7

142. Liang Z, Wu Q, Chen B, Yu P, Zhao H, Ouyang X. Effect of laparoscopic Roux-en-Y gastric bypass surgery on type 2 diabetes mellitus with hypertension: a randomized controlled trial. Diabetes Res Clin Pract. 2013;101(1):50–56. doi: 10.1016/j.diabres.2013.04.005

143. Liu J, Liu J, Fang D, Liu L, Huang Z, Wan X et al. Fasting plasma glucose after intensive insulin therapy predicted long-term glycemic control in newly diagnosed type 2 diabetic patients. Endocr J. 2013;60(6):725–732. doi: 10.1507/endocrj.ej12-0315

144. Liu L, Ke W, Wan X, Zhang P, Cao X, Deng W et al. Insulin requirement profiles of short-term intensive insulin therapy in patients with newly diagnosed type 2 diabetes and its association with long-term glycemic remission. Diabetes Res Clin Pract. 2015;108(2):250–257. doi: 10.1016/j.diabres.2015.02.011

145. Mathew L, Kilimozhi D, Parimala Krishnan S, Ismail M. Effect of metabolic surgery on type 2 diabetes remission: A matched group analysis. Int J Pharm Pharm Sci. 2015;7(9):206–209.

146. Mathew L, Kilimozhi D, Parimala Krishnan S, Ismail M. Metabolic effects of three different bariatric procedures-a retrospective study. Int J Pharm Pharm Sci. 2015;7(11):278–282.

147. Mu PW, Chen YM, Lu HY, Wen XQ, Zhang YH, Xie RY et al. Effects of a combination of oral anti-diabetes drugs with basal insulin therapy on beta-cell function and glycaemic control in patients with newly diagnosed type 2 diabetes. Diabetes Metab Res Rev. 2012;28(3):236–240. doi: 10.1002/dmrr.1292

148. Musella M, Susa A, Greco F, De Luca M, Manno E, Di Stefano C et al. The laparoscopic mini-gastric bypass: the Italian experience: outcomes from 974 consecutive cases in a multicenter review. Surg Endosc. 2014;28(1):156–163. doi: 10.1007/s00464-013-3141-y

149. Musella M, Apers J, Rheinwalt K, Ribeiro R, Manno E, Greco F et al. Efficacy of Bariatric Surgery in Type 2 Diabetes Mellitus Remission: the Role of Mini Gastric Bypass/One Anastomosis Gastric Bypass and Sleeve Gastrectomy at 1 Year of Follow-up. Obes Surg. 2016;26(5):933–940. doi: 10.1007/s11695-015-1865-6

150. Naitoh T, Kasama K, Seki Y, Ohta M, Oshiro T, Sasaki A et al. Efficacy of Sleeve Gastrectomy with Duodenal-Jejunal Bypass for the Treatment of Obese Severe Diabetes Patients in Japan: a Retrospective Multicenter Study. Obes Surg. 2018;28(2):497–505. doi: 10.1007/s11695-017-2874-4

151. Ng J, Seip R, Stone A, Ruano G, Tishler D, Papasavas P. Ethnic variation in weight loss, but not co-morbidity remission, after laparoscopic gastric banding and Roux-en-Y gastric bypass. Surg Obes Relat Dis. 2015;11(1):94–100.

152. Nor Hanipah Z, Hsin MC, Liu CC, Huang CK. Laparoscopic loop duodenaljejunal bypass with sleeve gastrectomy in type 2 diabetic patients. Surg Obes Relat Dis. 2019;15(5):696–702. doi: 10.1016/j.soard.2019.01.016

153. O'Rourke RW, Johnson GS, Purnell JQ, Courcoulas AP, Dakin GF, Garcia L, et al. Serum biomarkers of inflammation and adiposity in the LABS cohort: associations with metabolic disease and surgical outcomes. Int J Obes. 2019;43(2):285-96. doi: 10.1038/s41366-018-0088-z. PubMed PMID: 29777230; PubMed Central PMCID: PMCPMC6240401.

154. Panunzi S, Carlsson L, De Gaetano A, Peltonen M, Rice T, Sjostrom L et al. Determinants of Diabetes Remission and Glycemic Control After Bariatric Surgery. Diabetes Care. 2016;39(1):166–174. doi: 10.2337/dc15-0575

155. Park JY, Kim YJ. Prediction of Diabetes Remission in Morbidly Obese Patients After Roux-en-Y Gastric Bypass. Obes Surg. 2016;26(4):749–756.

156. Park JY, Kim YJ. Laparoscopic Roux-en-Y gastric bypass in obese Korean patients: efficacy and potential adverse events. Surg Today. 2016;46(3):348–355. doi: 10.1007/s00595-015-1170-y

157. Pessoa BM, Browning MG, Mazzini GS, Wolfe L, Kaplan A, Khoraki J et al. Factors Mediating Type 2 Diabetes Remission and Relapse after Gastric Bypass Surgery. J Am Coll Surg. 2020;230(1):7–16. doi: 10.1016/j.jamcollsurg.2019.09.012

158. Pucci A, Tymoszuk U, Cheung WH, Makaronidis JM, Scholes S, Tharakan G et al. Type 2 diabetes remission 2 years post Roux-en-Y gastric bypass and sleeve gastrectomy: the role of the weight loss and comparison of DiaRem and DiaBetter scores. Diabet Med. 2018;35(3):360–367. doi: 10.1111/dme.13532

159. Purnell JQ, Selzer F, Wahed AS, Pender J, Pories W, Pomp A et al. Type 2 Diabetes Remission Rates After Laparoscopic Gastric Bypass and Gastric Banding: Results of the Longitudinal Assessment of Bariatric Surgery Study. Diabetes Care. 2016;39(7):1101–1107. doi: 10.2337/dc15-2138

160. Ramos-Levi AM, Matia P, Cabrerizo L, Barabash A, Sanchez-Pernaute A, Calle-Pascual AL et al. Statistical models to predict type 2 diabetes remission after bariatric surgery. J Diabetes. 2014;6(5):472–477. doi: 10.1111/1753-0407.12127

161. Ramos-Levi AM, Sanchez-Pernaute A, Cabrerizo L, Matia P, Barabash A, Hernandez C et al. Remission of type 2 diabetes mellitus should not be the foremost goal after bariatric surgery. Obes Surg. 2013;23(12):2020–2025.

162. Ruiz-Tovar J, Carbajo MA, Jimenez JM, Castro MJ, Gonzalez G, Ortiz-de-Solorzano J et al. Long-term follow-up after sleeve gastrectomy versus Roux-en-Y gastric bypass versus one-anastomosis gastric bypass: a prospective randomized comparative study of weight loss and remission of comorbidities. Surg Endosc. 2019;33(2):401–410. doi: 10.1007/s00464-018-6307-9

163. Samuel N, Jalal Q, Gupta A, Mazari F, Vasas P, Balachandra S. Mid-term bariatric surgery outcomes for obese patients: does weight matter? Ann R Coll Surg Engl. 2020;102(1):54–61. doi: 10.1308/rcsann.2019.0100

164. Santoro S, Castro LC, Velhote MC, Malzoni CE, Klajner S, Castro LP et al. Sleeve gastrectomy with transit bipartition: a potent intervention for metabolic syndrome and obesity. Ann Surg. 2012;256(1):104–110. doi: 10.1097/SLA.0b013e31825370c0

165. Scally CP, Varban OA, Carlin AM, Birkmeyer JD, Dimick JB. Michigan Bariatric Surgery C. Video Ratings of Surgical Skill and Late Outcomes of Bariatric Surgery. JAMA Surg. 2016;151(6):e160428. doi: 10.1001/jamasurg.2016.0428

166. Schwoerer A, Kasten K, Celio A, Pories W, Spaniolas K. The effect of close postoperative follow-up on co-morbidity improvement after bariatric surgery. Surg Obes Relat Dis. 2017;13(8):1347–1352. doi: 10.1016/j.soard.2017.03.024

167. Seki Y, Kasama K, Haruta H, Watanabe A, Yokoyama R, Porciuncula JP et al. Five-Year-Results of Laparoscopic Sleeve Gastrectomy with Duodenojejunal Bypass for Weight Loss and Type 2 Diabetes Mellitus. Obes Surg. 2017;27(3):795–801. doi: 10.1007/s11695-016-2372-0

168. Sepulveda M, Alamo M, Preiss Y, Valderas JP. Metabolic Surgery Comparing Sleeve Gastrectomy with Jejunal Bypass and Roux-en-Y Gastric Bypass in Type 2 Diabetic Patients After 3 Years. Obes Surg. 2018;28(11):3466–3473. doi: 10.1007/s11695-018-3402-x

169. Ser KH, Lee WJ, Chen JC, Tsai PL, Chen SC, Lee YC. Laparoscopic single-anastomosis duodenal-jejunal bypass with sleeve gastrectomy (SADJB-SG): Surgical risk and long-term results. Surg Obes Relat Dis. 2019;15(2):236–243. doi: 10.1016/j.soard.2018.11.020

170. Shah K, Johnny Nergard B, Stray Frazier K, Geir Leifsson B, Aghajani E, Gislason H. Long-term effects of laparoscopic Roux-en-Y gastric bypass on metabolic syndrome in patients with morbid obesity. Surg Obes Relat Dis. 2016;12(8):1449–1456. doi: 10.1016/j.soard.2016.03.017

171. Shen SC, Wang W, Tam KW, Chen HA, Lin YK, Wang SY et al. Validating Risk Prediction Models of Diabetes Remission After Sleeve Gastrectomy. Obes Surg. 2019;29(1):221–229. doi: 10.1007/s11695-018-3510-7

172. Shi X, Shi Y, Chen N, Lin M, Su W, Zhang H et al. Effect of exenatide after short-time intensive insulin therapy on glycaemic remission maintenance in type 2 diabetes patients: a randomized controlled trial. Sci Rep. 2017;7(1):2383. doi: 10.1038/s41598-017-02631-1

173. Sjoholm K, Pajunen P, Jacobson P, Karason K, Sjostrom CD, Torgerson J et al. Incidence and remission of type 2 diabetes in relation to degree of obesity at baseline and 2 year weight change: the Swedish Obese Subjects (SOS) study. Diabetologia. 2015;58(7):1448–1453. doi: 10.1007/s00125-015-3591-y

174. Sjostrom L, Peltonen M, Jacobson P, Ahlin S, Andersson-Assarsson J, Anveden A et al. Association of bariatric surgery with long-term remission of type 2 diabetes and with microvascular and macrovascular complications. JAMA. 2014;311(22):2297–2304. doi: 10.1001/jama.2014.5988

175. Souteiro P, Belo S, Magalhaes D, Pedro J, Neves JS, Oliveira SC et al. Long-term diabetes outcomes after bariatric surgery-managing medication withdrawl. Int J Obes. 2019;43(11):2217–2224. doi: 10.1038/s41366-019-0320-5

176. Souteiro P, Belo S, Neves JS, Magalhaes D, Silva RB, Oliveira SC et al. Preoperative Beta Cell Function Is Predictive of Diabetes Remission After Bariatric Surgery. Obes Surg. 2017;27(2):288–294. doi: 10.1007/s11695-016-2300-3

177. Still CD, Benotti P, Mirshahi T, Cook A, Wood GC. DiaRem2: Incorporating duration of diabetes to improve prediction of diabetes remission after metabolic surgery. Surg Obes Relat Dis. 2019;15(5):717–724. doi: 10.1016/j.soard.2018.12.020

178. Sudan R, Jain-Spangler K. Tailoring Bariatric Surgery: Sleeve Gastrectomy, Roux-en-Y Gastric Bypass and Biliopancreatic Diversion with Duodenal Switch. J Laparoendosc Adv Surg Tech A. 2018;28(8):956–961. doi: 10.1089/lap.2018.0397

179. Sudan R, Maciejewski ML, Wilk AR, Nguyen NT, Ponce J, Morton JM. Comparative effectiveness of primary bariatric operations in the United States. Surg Obes Relat Dis. 2017;13(5):826–834. doi: 10.1016/j.soard.2017.01.021

180. Sundbom M, Hedberg J, Marsk R, Boman L, Bylund A, Hedenbro J et al. Substantial Decrease in Comorbidity 5 Years After Gastric Bypass: A Population-based Study From the Scandinavian Obesity Surgery Registry. Ann Surg. 2017;265(6):1166–1171. doi: 10.1097/SLA.0000000000001920

181. Taha O, Abdelaal M, Abozeid M, Askalany A, Alaa M. Outcomes of One Anastomosis Gastric Bypass in 472 Diabetic Patients. Obes Surg. 2017;27(11):2802–2810. doi: 10.1007/s11695-017-2711-9

182. Taylor BA, Ng J, Stone A, Thompson PD, Papasavas PK, Tishler DS. Effects of statin therapy on weight loss and diabetes in bariatric patients. Surg Obes Relat Dis. 2017;13 (4):674–680. doi: 10.1016/j.soard.2016.11.018

183. Tharakan G, Scott R, Szepietowski O, Miras AD, Blakemore AI, Purkayastha S et al. Limitations of the DiaRem Score in Predicting Remission of Diabetes Following Roux-En-Y Gastric Bypass (RYGB) in an ethnically Diverse Population from a Single Institution in the UK. Obes Surg. 2017;27(3):782–786. doi: 10.1007/s11695-016-2368-9

184. Thereaux J, Corigliano N, Poitou C, Oppert JM, Czernichow S, Bouillot JL. Comparison of results after one year between sleeve gastrectomy and gastric bypass in patients with BMI >/= 50 kg/m(2). Surg Obes Relat Dis. 2015;11(4):785–790. doi: 10.1016/j.soard.2014.11.022

185. Toh BC, Chan WH, Eng AKH, Lim EKW, Lim CH, Tham KW et al. Five-year long-term clinical outcome after bariatric metabolic surgery: A multi-ethnic Asian population in Singapore. Diabetes Obes Metab. 2018;20(7):1762–1765. doi: 10.1111/dom.13263

186. Van De Laar AW, De Brauw LM, Meesters EW. Relationships between type 2 diabetes remission after gastric bypass and different weight loss metrics: Arguments against excess weight loss in metabolic surgery. Surg Obes and Relat Dis. 2016;12(2):274–282.

187. Velazquez-Fernandez D, Sanchez H, Monraz F, Zanela OO, Cabra HA, Pantoja JP et al. Development of an Interactive Outcome Estimation Tool for Laparoscopic Roux-en-Y Gastric Bypass in Mexico Based on a Cohort of 1002 Patients. Obes Surg. 2019;29(9):2878–2885. doi: 10.1007/s11695-019-03929-x

188. Victorzon M, Peromaa-Haavisto P, Tolonen P. Perioperative morbidity, mortality and early outcome of the first 360 gastric bypass operations performed in a district hospital. Scand J Surg. 2012;101(3):184–189. doi: 10.1177/145749691210100308

189. Viscido G, Gorodner V, Signorini FJ, Biasoni AC, Navarro L, Rubin G et al. Obese Patients with Type 2 Diabetes: Outcomes After Laparoscopic Sleeve Gastrectomy. J Laparoendosc Adv Surg Tech A. 2019;29(5):655–662. doi: 10.1089/lap.2018.0652

190. Wadden TA, Chao AM, Bahnson JL, Bantle JP, Clark JM, Gaussoin SA et al. End-of-Trial Health Outcomes in Look AHEAD Participants who Elected to have Bariatric Surgery. Obesity (Silver Spring). 2019;27(4):581–590. doi: 10.1002/oby.22411

191. Walker E, Elman M, Takemoto EE, Fennern E, Mitchell JE, Pories WJ et al. Bariatric Surgery Among Medicare Subgroups: Short- and Long-Term Outcomes. Obesity (Silver Spring). 2019;27(11):1820–1827. doi: 10.1002/oby.22613

192. Wazir N, Arshad MF, Finney J, Kirk K, Dewan S. Two Years Remission of Type 2 Diabetes Mellitus after Bariatric Surgery. J Coll Physicians Surg Pak. 2019;29 (10):967–971. doi: 10.29271/jcpsp.2019.10.967

193. Wei JH, Chou RH, Huang PH, Lee WJ, Chen SC, Lin SJ. Metabolic surgery ameliorates cardiovascular risk in obese diabetic patients: Influence of different surgical procedures. Surg Obes Relat Dis. 2018;14(12):1832–1840. doi: 10.1016/j.soard.2018.08.026

194. Wood GC, Gerhard GS, Benotti P, Petrick AT, Gabrielsen JD, Strodel WE et al. Preoperative use of incretins is associated with increased diabetes remission after RYGB surgery among patients taking insulin: a retrospective cohort analysis. Ann Surg. 2015;261(1):125–128. doi: 10.1097/SLA.0000000000000588

195. Wood MH, Carlin AM, Ghaferi AA, Varban OA, Hawasli A, Bonham AJ et al. Association of Race With Bariatric Surgery Outcomes. JAMA Surg. 2019;154(5):e190029. doi: 10.1001/jamasurg.2019.0029

196. Xu L, Wang L, Huang X, Liu L, Ke W, He X et al. Baseline red blood cell distribution width predicts long-term glycemic remission in patients with type 2 diabetes. Diabetes Res Clin Pract. 2017;131:33–41. doi: 10.1016/j.diabres.2017.06.019

197. Yan W, Bai R, Li Y, Xu J, Zhong Z, Xing Y et al. Analysis of Predictors of Type 2 Diabetes Mellitus Remission After Roux-en-Y Gastric Bypass in 101 Chinese Patients. Obes Surg. 2019;29(6):1867–1873. doi: 10.1007/s11695-019-03783-x

198. Young L, Nor Hanipah Z, Brethauer SA, Schauer PR, Aminian A. Long-term impact of bariatric surgery in diabetic nephropathy. Surg Endosc. 2019;33(5):1654–1660. doi: 10.1007/s00464-018-6458-8

199. Zaman JA, Shah N, Leverson GE, Greenberg JA, Funk LM. The effects of optimal perioperative glucose control on morbidly obese patients undergoing bariatric surgery. Surg Endosc. 2017;31(3):1407–1413. doi: 10.1007/s00464-016-5129-x

200. Zaveri H, Surve A, Cottam D, Cottam A, Medlin W, Richards C et al. Mid-term 4-Year Outcomes with Single Anastomosis Duodenal-Ileal Bypass with Sleeve Gastrectomy Surgery at a Single US Center. Obes Surg. 2018;28(10):3062–3072. doi: 10.1007/s11695-018-3358-x

201. Zenti MG, Rubbo I, Ceradini G, Rinaldi E, Nadalini L, Battistoni M, et al. Clinical factors that predict remission of diabetes after different bariatric surgical procedures: interdisciplinary group of bariatric surgery of Verona (G.I.C.O.V.). Acta Diabetol 2015;52(5):937-42. doi: 10.1007/s00592

202. Zhang H, Di J, Yu H, Han X, Li K, Zhang P. The Short-Term Remission of Diabetic Nephropathy After Roux-en-Y Gastric Bypass in Chinese Patients of T2DM with Obesity. Obes Surg. 2015;25(7):1263–1270. doi: 10.1007/s11695-015-1666-y

203. Zhang H, Han X, Yu H, Di J, Zhang P, Jia W. Effect of Roux-en-Y Gastric Bypass on Remission of T2D: Medium-Term Follow-up in Chinese Patients with Different BMI Obesity Class. Obes Surg. 2017;27(1):134–142. doi: 10.1007/s11695-016-2262-5

204. Zhu Z, Shan X, Cheng Y, Xu J, Fu H, Wang W et al. Clinical course of diabetes after gastrectomy according to type of reconstruction in patients with concurrent gastric cancer and type 2 diabetes. Obes Surg. 2015;25(4):673–679. doi: 10.1007/s11695-014-1426-4
