# Supplementary material for: Defining remission of type 2 diabetes in research studies: A systematic scoping review
Source: PLoS Med. 2020 Oct 28;17(10):e1003396. doi: 10.1371/journal.pmed.1003396 (PMC7592769; doi:10.1371/journal.pmed.1003396)
Supplement: S5 Table — (DOCX) [file pmed.1003396.s009.docx]

**S5 Table. Combinations of numeric glycaemic parameters used to signify normoglycaemia for all definitions of remission (206 definitions included glycaemic parameters)**

| HbA1c (mmol/mol) | Definition **ONLY** based on HbA1c  n (%) | Fasting Plasma Glucose (FPG) (mmol/l) | | | | | | | | | | |
| --- | --- | --- | --- | --- | --- | --- | --- | --- | --- | --- | --- | --- |
|  |  | Definition based on HbA1c **AND** FPG | | | Definition based on HbA1c **OR** FPG | | Definition based on HbA1c**,** FPG **association not clear^g^** | | | Definition **ONLY** based on FPG | | |
|  |  | <7.0  n (%) | <6.0  n (%)^d^ | <5.6  n (%) | <7.0  n (%) | <5.6  n (%) | <7.0  n (%) | <6.0  n (%) | <5. 6  n (%) | <7.0  n (%) | <6.0  n (%)^d^ | <5.60  n (%) |
| Definition ONLY based on FPG (n=10) | **-** | - | - | - | - | - | - | - | - | 1 (0.4) | 2 (0.9) | 7 (3.0) |
| <53 (n=4) | 3 (1.3) |  |  |  | 1 (0.4) |  |  |  |  | - | - | - |
| <48 (n=103) | 45 (19.4)^a^ | 30 (12.9) | 1 (0.4) | 3 (1.3) | 9 (3.9) |  | 13 (5.6) |  | 2 (0.9) | - | - | - |
| <42 (n=89) | 47 (20.3) | 2 (0.9) | 5 (2.2) | 24 (10.3) |  | 3 (1.3) |  | 1 (0.4) | 7 (3.0) | - | - | - |
| <39 (n=19) | 8 (3.4) |  |  | 10 (4.3) |  | 1 (0.4) |  |  |  | - | - | - |
| Other (n=7) | 2 (0.9)^b^ | 3 (1.3)^c^ | 1 (0.4)^e^ |  | 1(0.4)^f^ |  |  |  |  | - | - | - |
| Total (n=232) | 105(45.3) | 35 (15.1) | 7 (3.0) | 37 (15.9) | 11 (4.7) | 4 (1.7) | 13(5.6) | 1 (0.4) | 9 (3.9) | 1 (0.4) | 2 (0.9) | 7 (3.0) |

a one study used HbA1c<48mmol/mol but also defined remission if no HbA1c readings were taken within a given year

b one study used HbA1c <46mmol/mol, one study used HbA1c<42mmol/mol with GLT OR HbA1c<48 without GLT

c three studies used 2 hr PG<10.0mmol/l AND FPG<7.0mmol/l

d including studies that also specified FPG <6.1mmol/l

e one study used 2 hr PG<8.0mmol/l AND FPG<6.1mmol/l

f one study used 2-hr PG<8.0mmol/l OR FPG<7.0mmol/l

g Ambiguous association between HbA1c and FPG, not possible to categories as AND or OR e.g. HbA1c <48mmol/mol, FPG<7.0mmol/l
