# Supplementary material for: Defining remission of type 2 diabetes in research studies: A systematic scoping review
Source: PLoS Med. 2020 Oct 28;17(10):e1003396. doi: 10.1371/journal.pmed.1003396 (PMC7592769; doi:10.1371/journal.pmed.1003396)
Supplement: S6 Table — (DOCX) [file pmed.1003396.s010.docx]

**S6 Table: Possible interpretations of the 2009 Report**

| Remission term | Glucose lowering therapy (GLT) | Glycaemic Element | | | Time (years) |
| --- | --- | --- | --- | --- | --- |
|  |  | HbA1c (mmol/mol) | Association | Fasting Plasma Glucose (FPG) (mmol/l) |  |
| Remission | No GLT | <48 | AND | 5.6-6.9 | 1 |
|  |  | <48 | OR | 5.6-6.9 |  |
|  |  | “normal” | AND | <5.6 |  |
|  |  | “normal” | OR | <5.6 |  |
|  |  | <42 | AND | <5.6 |  |
|  |  | <42 | OR | <5.6 |  |
|  |  | <39 | AND | <5.6 |  |
|  |  | <39 | OR | <5.6 |  |
| Remission | No GLT | <48 | AND | 5.6-6.9 | Any |
|  |  | <48 | OR | 5.6-6.9 |  |
|  |  | “normal” | AND | <5.6 |  |
|  |  | “normal” | OR | <5.6 |  |
|  |  | <42 | AND | <5.6 |  |
|  |  | <42 | OR | <5.6 |  |
|  |  | <39 | AND | <5.6 |  |
|  |  | <39 | OR | <5.6 |  |
| Partial Remission | No GLT | <48 | AND | 5.6-6.9 | 1 |
|  |  | <48 | OR | 5.6-6.9 |  |
| Complete  Remission | No GLT | “normal” | AND | <5.6 | 1 |
|  |  | “normal” | OR | <5.6 |  |
|  |  | <42 | AND | <5.6 |  |
|  |  | <42 | OR | <5.6 |  |
|  |  | <39 | AND | <5.6 |  |
|  |  | <39 | OR | <5.6 |  |
|  | No GLT | “normal” | AND | <5.6 | 5 |
|  |  | “normal” | OR | <5.6 |  |
|  |  | <42 | AND | <5.6 |  |
|  |  | <42 | OR | <5.6 |  |
|  |  | <39 | AND | <5.6 |  |
|  |  | <39 | OR | <5.6 |  |
