# Supplementary material for: Defining remission of type 2 diabetes in research studies: A systematic scoping review
Source: PLoS Med. 2020 Oct 28;17(10):e1003396. doi: 10.1371/journal.pmed.1003396 (PMC7592769; doi:10.1371/journal.pmed.1003396)
Supplement: S7 Table — (DOCX) [file pmed.1003396.s011.docx]

**S7 Table. Definitions of remission organised by unspecified remission, partial remission, complete remission and prolonged remission.**

| GLT component | Glycaemic Component | Time Comp- onent | n | Study |
| --- | --- | --- | --- | --- |
| Remission Unspecified | | | | |
| Absence of GLT specified | HbA1c <39mmol/mol (5.7%) & FPG <5.6mmol/l (100mg/dl) | For 1 year | 1 | [36] |
|  | HbA1c <39mmol/mol (5.7%) |  | 2 | [38, 114] |
|  | HbA1c <42mmol/mol (6.0%), FPG <5.6mmol/l (100mg/dL) |  | 1 | [60] |
|  | HbA1c <42mmol/mol (6.0%) |  | 2 | [69, 137] |
|  | HbA1c <48mmol/mol (6.5%) & FPG <7.0mmol/l (126mg/dL) |  | 4 | [36, 122, 123, 202] |
|  | HbA1c<48mmol/mol (6.0%), FPG <7.0mmol/l (126mg/dL) |  | 1 | [55] |
|  | HbA1c<48mmol/mol (6.5%) |  | 4 | [48, 98, 105, 184] |
|  | HbA1c<48mmol/mol (6.5%) after 1 year of no GLT (or no HbA1c testing in the year) |  | 1 | [157] |
|  | HbA1c<48mmol/mol (6.5%) or FPG <7.0mmol/l (126mg/dL) |  | 1 | [149] |
|  | FPG<7.0mmol/l (126mg/dL) or 2-hr PG <10.0mmol/l (180mg/dL) |  | 1 | [172] |
|  | FPG<7.0mmol/l (126mg/dL) and 2-hr PG <10.0mmol/l (180mg/dL) |  | 2 | [143, 196] |
|  | FPG<6.1 mmol/l (110mg/dL) & 2-hr PG <8.0mmol/l (144mg.dL) (after 3 meals) |  | 1 | [147] |
|  | "Normoglycaemia" |  | 2 | [67, 83] |
|  | HbA1c <42mmol/mol (6.0%) | For 6 months | 1 | [30] |
|  | HbA1c<48mmol/mol (6.5%) |  | 2 | [31, 32] |
|  | First HbA1c<48mmol/mol (6.5%) (after 6 m of no GLT) |  | 1 | [37] |
|  | missing |  | 1 | [33] |
|  | HbA1c <53mmol/mol (7.0%) or FBG <7.0mmol/l (126mg/dl) | for 90 days | 1 | [34] |
|  | HbA1c <48mmol/mol (6.5%) | for 2 months^a^ | 1 | [13] |
|  | HbA1c <39mmol/mol (5.7%) and FPG<5.6mmol/l (100mg/dL) | For 30 days | 1 | [36] |
|  | HbA1c<48mmol/mol (6.5%) and FPG <7.0mmol/l (126 mg/dL) |  | 1 | [36] |
|  | HbA1c<48mmol/mol (6.5%) after 1 year of no GLT | Within 2 time points | 1 | [157] |
|  | HbA1c <39mmol/mol (5.7%) | at 1 year | 1 | [42] |
|  | HbA1c <42mmol/mol (6.0%) or FPG <5.6mmol/l (100mg/dL) |  | 1 | [9] |
|  | HbA1c <42mmol/mol (6.0%) and FPG <6.0mmol/l (108mg/dL) |  | 1 | [111] |
|  | HbA1c <42mmol/mol (6.0%) and FPG <7.0mmol/l (126mg/dL) |  | 2 | [91, 182] |
|  | HbA1c <42mmol/mol (6.0%) |  | 2 | [42, 186] |
|  | HbA1c <48mmol/mol |  | 5 | [42, 95, 99, 185, 199] |
|  | HbA1c <53 mmol/mol [42](7.0%) |  | 1 | [42] |
|  | missing |  | 3 | [119, 165, 195] |
|  | missing | at 18 months | 1 | [110] |
|  | FPG <5.6mmol/l (100mg/dL) | at 2 years | 1 | [154] |
|  | HbA1c <39mmol/mol (5.7%) and FPG <5.6mmol/l (100mg/dL) | missing | 2 | [36, 41] |
|  | HbA1c <39mmol/mol (5.7%) |  | 1 | [41] |
|  | HbA1c <42mmol/mol (6.0%) and FPG <5.6mmol/l (100mg/dL) |  | 6 | [41, 117, 125, 160, 161, 201] |
|  | HbA1c <42mmol/mol (6.0%) and FPG <6.0mmol/l (108mg/dL) |  | 1 | [24] |
|  | HbA1c <42mmol/mol (6.0%), FPG <6.1mmol/l (110mg/dL) |  | 1 | [56] |
|  | HbA1c <42mmol/mol (6.0%) |  | 7 | [41, 79, 81, 127, 133, 167, 188] |
|  | HbA1c<48mmol/mol (6.5%) and FPG <7.0mmol/l (126 mg/dL) |  | 5 | [36, 70, 89, 126, 156] |
|  | HbA1c<48mmol/mol (6.5%) and FPG <7.0mmol/l (126 mg/dL) and 2hr PG <11.1mmol/l (<200mg/dL) |  | 1 | [103] |
|  | HbA1c<48mmol/mol (6.5%), FPG <7.0mmol/l (126 mg/dL) |  | 2 | [59, 71] |
|  | HbA1c<48mmol/mol (6.5%) and FPG <6.1mmol/l (110 mg/l) |  | 1 | [162] |
|  | HbA1c<48mmol/mol (6.5%) |  | 7 | [88, 106, 107, 130, 131, 148, 164] |
|  | HbA1c<48mmol/mol (6.5) or FPG <7.0mmol/l (126 mg/dL) |  | 5 | [47, 153, 159, 192, 198] |
|  | HbA1c <53mmol/mol (7%) |  | 1 | [128] |
|  | FPG <6.1mmol/l (110mg/dL) |  | 2 | [173, 174] |
|  | FPG <5.6mmol/l (100mg/dL) |  | 4 | [41, 82, 166, 204] |
|  | FPG <7.0mmol/l (126mg/dL) and 2-hr PG <10mmol/L (180mg/dL) |  | 1 | [144] |
|  | "Normoglycaemia" |  | 6 | [68, 86, 97, 112, 124, 187] |
|  | no symptoms or evidence of diabetes |  | 3 | [74, 85, 151] |
|  | Missing |  | 8 | [65, 76, 90, 101, 120, 178-180] |
| Absence of GLT except Metformin | HbA1c <42mmol/mol (6.0%) | missing | 1 | [26] |
| Absence of GLT or  Metformin | HbA1c<48mmol/mol (6.5%) (without metformin) or 42mmol/mol (6.0%) (with metformin) | for 6 months | 1 | [23] |
| missing | HbA1c <42mmol/mol | at 1 year | 1 | [94] |
|  | HbA1c <39mmol/mol (5.7%) and FPG <5.6 (100mg/dL) | missing | 1 | [102] |
|  | Absence of diabetes | missing^b^ | 2 | [87, 191] |
|  | ADA definition of remission | missing | 2 | [116, 163] |
| **Partial remission** | | | | |
| Absence of GLT stated | HbA1c <48mmol/mol (6.5%) & FPG <7.0mmol/l (126 mg/dL) | for 1 year | 10 | [38, 39, 80, 84, 92, 170, 177, 183, 189, 194] |
|  | HbA1c<48mmol/mol (6.5%), FPG <7.0mmol/l (126 mg/dL) |  | 4 | [50, 54, 57, 58, 71] |
|  | HbA1c < 48mmol/mol (6.0%) |  | 9 | [39, 118, 132, 138, 140, 158, 169, 181, 193] |
|  | HbA1c <46mmol/mol (6.4%) |  | 1 | [21] |
|  | HbA1c<53mmol/mol (7.0%) |  | 1 | [98] |
|  | FPG <7.0mmol/l (126mg/dL) |  | 1 | [134] |
|  | HbA1c <48mmol/mol (6.5%) or FPG <7.0mmol/l (126mg/dL) | for 90 days | 1 | [35] |
|  | HbA1c<48mmol/mol (6.5%) & FPG <7.0mmol/l (126 mg/dL) | at 1 year | 2 | [9, 115] |
|  | HbA1c<48mmol/molv (6.5%), FPG <7.0mmol/l (126 mg/dL) |  | 1 | [51] |
|  | HbA1c <48mmol/mol (6.5%) |  | 2 | [146, 150] |
|  | HbA1c<48mmol/mol (6.5%) | at 18 months | 1 | [121] |
|  | HbA1c<48mmol/mol (6.5%) & FPG <7.0mmol/l (126 mg/dL) | missing | 5 | [14, 49, 66, 100, 109] |
|  | HbA1c<48mmol/mol (6.5%) or FPG <7.0mmol/l (126 mg/dL) |  | 1 | [129] |
|  | HbA1c<48mmol/mol (6.5%), FPG <7.0mmol/l (126 mg/dL) |  | 2 | [40, 52] |
|  | HbA1c<48mmol/mol (6.5%) |  | 8 | [75, 77, 135, 152, 156, 171, 175, 190] |
| “Some GLT” | HbA1c <48mmol/mol (6.5) | at 1 year | 2 | [27, 28] |
|  | Achievement of ADA criteria | for 6 months | 1 | [25] |
|  | HbA1c <48mmol/mol (6.5%) OR FPG<7mmol/l (126mg/dL) (metformin allowed if taken for non-T2D indication) | missing | 1 | [29] |
|  | Normoglycaemia |  | 1 | [24] |
| GLT missing | HbA1c <42mmol/mol (6.0%) | at 1 year | 1 | [78] |
|  | HbA1c<48mmol/mol (6.5%) | missing | 1 | [136] |
|  | “2009 consensus ADA criteria” |  | 1 | [93] |
| **Complete remission** | | | | |
| Absence of GLT stated | HbA1c <39mmol/mol (5.7%) & FPG <5.6mmol/l (100mg/dL) | for 1 year | 3 | [176, 177, 194] |
|  | HbA1c <39mmol/mol (5.7%) |  | 2 | [21, 39] |
|  | HbA1c <42mmol/mol (6.0%) & FPG <5.6mmol/l (100mg/dL) |  | 7 | [38, 80, 84, 92, 183, 189, 203] |
|  | HbA1c <42mmol/mol (6.0%), FPG <5.6mmol/l (100mg/dL) |  | 2 | [57, 58] |
|  | HbA1c <42mmol/mol (6.0%) |  | 8 | [118, 132, 138, 140, 158, 169, 181, 193] |
|  | HbA1c <48mmol/mol (6.5%) & FPG <5.6mmol/l (100mg/dL) |  | 2 | [39, 197] |
|  | FPG <5.6mmol/l (100mg/dL) |  | 1 | [134] |
|  | Achievement of ADA criteria | for 6 months | 1 | [25] |
|  | HbA1c <42mmol/mol (6.0%) or FPG <5.6mmol/l (100mg/d/L) | for 90 days | 1 | [35] |
|  | HbA1c <42mmol/mol (6.0%) & FPG <5.6mmol/l (100mg/dL) | at 1 year | 2 | [9, 170] |
|  | HbA1c <42mmol/mol (6.0%), FPG <5.6mmol/l (100mg/dL) |  | 1 | [51] |
|  | Hba1c<42mmol/mol (6.0%) & FPG<6.1mmol/l (110mg/dl) |  | 1 | [115] |
|  | HbA1c<42mmol/mol (6.0%) |  | 7 | [27, 28, 108, 113, 145, 146, 150] |
|  | HbA1c <42mmol/mol (6.0%) | at 18 months | 1 | [121] |
|  | HbA1c < 39mmol/mol (5.7%) & FPG <5.6mmol/l (100mg/dL) | missing | 2 | [14, 100] |
|  | HbA1c<39mmol/mol |  | 1 | [190] |
|  | HbA1c < 42mmol/mol (6.0%) & FPG <5.6mmol/l (100mg/dL) |  | 5 | [49, 66, 96, 109, 168] |
|  | HbA1c <42mmol/mol (6.0%) or FPG <5.6mmol/l (100mg/dL) |  | 1 | [129] |
|  | HbA1c <42mmol/mol (6.0%), FPG <5.6mmol/l (<100mg/dL) |  | 2 | [40, 52] |
|  | HbA1c < 42mmol/mol (6.0%) and FPG <110 (6.1mg/dL) |  | 2 | [139, 141] |
|  | HbA1c <42mmol/mol (6.0%) |  | 8 | [75, 77, 135, 152, 155, 171, 175, 200] |
|  | HbA1c < 48mmol/mol (6.5%), FPG <5.6mmol/l (100mg/dL) |  | 1 | [40] |
| Some GLT | HbA1c <39mmol/mol (5.7%) OR FPG<5.6mmol/l (100mg/dL) (metformin allowed if taken for non-T2D indication) |  | 1 | [29] |
| missing | HbA1c <42mmol/mol (6.0%) |  | 1 | [136] |
|  | Defined according to 2009 consensus ADA criteria |  | 1 | [93] |
| Prolonged remission | | | | |
| Absence of GLT stated | HbA1c <39mmol/mol (5.7%) | for 5 years |  | [21] |
|  | HbA1c<42mmol/mol (6.0%) and FPG <5.6mmol/l (100mg/dL) |  | 2 | [73, 80] |
|  | HbA1c<42mmol/mol (6.0%), FPG <5.6mmol/l (100mg/dl) |  | 1 | [40] |
|  | HbA1c<42mmol/mol (6.0%) |  | 7 | [69, 75, 132, 137, 138, 169, 193] |
|  | HbA1c <48mmol/mol (6.5%) and FPG <5.6mmol/l (100mg/dL) |  | 1 | [39] |
|  | HbA1c <48mmol/mol (6.5%), FPG <5.6mmol/l (100mg/dL) |  | 1 | [40] |
|  | HbA1c<48mmol/mol (6.5%), FPG <7.0mmol/l (126 mg/dL) |  | 1 | [53] |
|  | FPG <5.6mmol/l (100mg/dL) |  | 1 | [134] |
|  | HbA1c<48mmol/mol (6.5%), FPG <7.0mmol/l (126 mg/dL) | at 5 years | 1 | [71, 72] |
|  | HbA1c<48mmol/mol (6.5%) and FPG <7.0mmol/l (126 mg/dL) |  | 2 | [73, 80] |
|  | HbA1c <42mmol/mol (6.0%) and FPG <5.6mmol/l (100 mg/dL) |  | 2 | [73, 80] |

a absence of GLT for at least 2 months, remission was diagnosed at 1 year

b at follow up
