# Supplementary material for: Defining remission of type 2 diabetes in research studies: A systematic scoping review
Source: PLoS Med. 2020 Oct 28;17(10):e1003396. doi: 10.1371/journal.pmed.1003396 (PMC7592769; doi:10.1371/journal.pmed.1003396)
Supplement: Attachment — Submitted filename: Response to reviewers.pdf [file pmed.1003396.s012.pdf]

Dr Artur Arikainen
Associate Editor
Plos Medicine

2nd August 2020

Dear Dr Arikainen,

Defining remission of type 2 diabetes in research studies: A systematic scoping review

Thank you for your interest in our paper. We have responded to the helpful comments from the reviewers and editor which we believe has strengthened the paper. For your convenience we reproduce the reviewers' comments verbatim, before detailing our response to each of the points made. Page and line numbers refer to the clean manuscript revision.

We hope our revised manuscript now meets with your approval. This manuscript has been seen and approved by each author. Please feel free to contact me if you require any further clarifications.

Your Sincerely,

Dr Mireille Captieux, on behalf of all authors.

Response to editors' comments:

1. Data Availability: At this stage, please make the raw extraction table available in a public repository or add the excel sheet to supplementary information file, as you suggest.

Response. We have added the raw excel extraction table to a public repository and can also submit as an "other file" if required.

2. Abstract:

a. Please report your abstract according to PRISMA for abstracts, following the PLOS Medicine abstract structure (Background, Methods and Findings, Conclusions)
- http://www.plosmedicine.org/article/info:doi/10.1371/journal.pmed.1001419.

Response. We have restructured the abstract according to PRISMA for abstracts following the PLOS Medicine structure as suggested. [page 3 line 21-51]

b. Please avoid “consensus” for the 2009 report, here and throughout the text, as recommended by reviewer #1.

Response. We agree that the nomenclature of the 2009 report published in Diabetes Care is confusing. We have explained this in the abstract [page 3 line 25-27] and modified all references to this publication from “2009 consensus report” to “2009 report” to clarify any doubt. [page 3 line 31 (and throughout manuscript)]

c. In the last sentence of the Abstract Methods and Findings section, please describe the main limitation(s) of the study's methodology.

Response. We have added the main limitation of this systematic review to the Abstract Methods and Findings section. [page 3, line 41-43]

3. At this stage, we ask that you include a short, non-technical Author Summary of your research to make findings accessible to a wide audience that includes both scientists and non-scientists. The Author Summary should immediately follow the Abstract in your revised manuscript. This text is subject to editorial change and should be distinct from the scientific abstract. Please see our author guidelines for more information: https://journals.plos.org/plosmedicine/s/revising-your-manuscript#loc-author-summary

Response. We have added an Author Summary in line with the PLOS Medicine guidance and understand the text is subject to editorial change. [Page 5 line 53-82]

4. Please use the "Vancouver" style for reference formatting, and see our website for other reference guidelines https://journals.plos.org/plosmedicine/s/submission-guidelines#loc-references. Citations should be in square brackets, and preceding punctuation.

Response. We have modified the reference formatting to “Vancouver” style as per the PLOS Medicine guidance.

5. Please avoid using italics for emphasis, eg. lines 66, 68, 274.

Response. We have removed italics at the specified points. [page 7 line 95, 98, p17 line 326]

6. Please remove colons from the ends of section and subsection headings, eg “Search strategy and selection criteria”.

Response. We have removed colons at the specified points. [page 10 line 147, page 17 line 298]

7. Please update your search, as it is now over 12 months old and relevant articles published in that time may have been missed.

Response. We have updated our search using the same strategy and reviewers as in the initial search. All title and abstract screening, full text screening and data extraction were performed independently in duplicate by two review authors. We identified 22 extra papers which matched inclusion criteria for addition to this systematic review. The systematic review is now up to date to 18th July 2020.

8. Please remove the funding information on lines 167-170. This is taken from the online submission form instead.

Response. We have removed the funding information as requested.

9. Please present and organize the Discussion as follows: a short, clear summary of the article's findings; what the study adds to existing research and where and why the results may differ from previous

research; strengths and limitations of the study; implications and next steps for research, clinical practice, and/or public policy; one-paragraph conclusion.

Response. We have re-organised the Discussion (without using subject headings) as requested. [page 17 line 299- 398]

10. Please provide a URL for reference 162.

Response. We have now updated this reference.

11. Please provide a copy of the completed PRISMA checklist. When completing the checklist, please use section and paragraph numbers, rather than page numbers.

Response. We have completed and uploaded an amended PRISMA checklist separately and in our supplementary information.

Comments from the reviewers:

Reviewer #1: The aims of this systematic review are very reasonable. Although the information distilled from the literature is unsurprising for those working in this area, the need for a widely accepted definition of remission of type 2 diabetes is not generally appreciated. Some inaccuracies require to be corrected.

1. In the Abstract the statement that there is 'no consensus' requires to be rephrased. In the UK, the Primary Care Diabetes Society and the Association of British Clinical Diabetologists have published their consensus (Nagi et al 2019, Brit J Diabetes 19: 73-76; https://www.pcdsociety.org/news/details/acbd-and-pcds-release-joint-position-statement-on-remission-of-type-2-diabetes) and the 2009 Diabetes care consensus is still widely used.

Response. We have rephrased the 'no consensus' statement in the abstract [page 3 line 23-25] and added the PCDS/ABCD UK consensus remission statement to our manuscript. [page 7 line 135-140]

2. The ADA neither commissioned nor formally accepted the 2009 consensus document. Reference to this document requires to be adjusted throughout. it was a consensus statement from a group of experts, published in an ADA Journal. Even though the authors correctly state this (line 72-74), the description elsewhere is inaccurate.

Response. We agree, this is a potentially confusing area. We have added a further clarification in the abstract page 3 [line 25-27], introduction [page 7, line 102] and now refer to the "2009 report" throughout the manuscript rather than the "2009 consensus report". [page 7 line 102]

3. Line 42: the definition of normal fasting plasma glucose differs in the US (5.6mmol/l) and the UK (6.1 mol/l). The wording should be amended accordingly.

Response. We originally used the general term normoglycaemia to refer to the multiple permutations of FPG that study authors chose to represent remission. These are outlined in the S5 table. However, as you quite rightly point out, this is unclear and there is insufficient space in the abstract to clarify what we mean. We therefore have updated the abstract to report the single most frequently used threshold (HbA1c<42mmol/mol (6.0%) alone reported in 47 definitions) (rather than the most frequently used HbA1c threshold in combination with multiple permutations of FPG thresholds (103 definitions used HbA1c in combination with different values of FPG. e.g. HbA1c <48mmol/mol (6.5%) AND FPG<6.1mmol/l (110mg/dL) HbA1c<48mmol/mol (6.5%) AND FPG <5.6mmol/l (100mg/dL), HbA1c<48mmol/mol (6.5%) OR FPG <6.1mmol/l (110mg/dL) etc.). We think this is clearer for the abstract and we discuss the different permutations of HbA1c, FPG and 2-hr PG in greater detail within the results section. [page 3 line 40]

4. Line 87. Undue emphasis appears to be given in the introduction to the 2009 expert group opinion. In a systematic review should one paper really monopolise the Introduction?

> **Response.** We initially focus on the 2009 report/expert group opinion in the introduction, because rightly or wrongly it is the publication that has most shaped the discussion of remission definition so far (reflected in the frequency with which studies of remission cite it). We felt that readers less familiar with remission would benefit from understanding where the terms "partial remission" "complete remission" and "prolonged remission" originated from, especially, as many of the research papers included in our research also adopted the same naming conventions. The definition of remission based on three components (absence of glucose lowering therapy, glycaemic threshold and duration) was introduced by the 2009 report and it is the structure we used to extract and analyse each definition of remission. We have rebalanced the introduction by adding in details of the PCDS/ABCD 2019 consensus and mentioned forthcoming guidance. [page 8 line 135-141]. We are happy to move Table 1 to supplementary material if it is felt that it does not add anything to the introduction.

5. Line 129. Involvement of people with diabetes is important in respect of the critical matter of the ease of understanding and using a definition in the care of individuals. At the very least, the James Lind Alliance / DUK survey of the most important research questions in diabetes should be quoted (Finer et al; Diabetic Medicine 2018:35:862-870). This has major implications for the patient centred need to have HbA1c rather than fasting or OGTT plasma glucose criteria due to how Primary Care operates. Reference to the james Lind Alliance's identification of remission as the number 1 important research question would be appropriate.

> **Response.** We have summarised the top findings from this survey and referenced the Diabetes-UK-James Lind Partnership as suggested within this section. [page 10 line 163-167] We also refer back to these survey findings in our conclusion [page 19 line 388-389]. We had referenced this publication in the introduction, however I agree it requires greater prominence in our manuscript, so thank you for bringing this to our attention.

6. Line 305 "identifies' should be replaced with 'supports'. This point takes account of the almost completed consensus discussions between ADA, EASD and DUK which are almost complete. The present SR is timely in respect of likely publication of International consensus guidelines next year.

> **Response.** We agree. This change makes it clear that this review supports the need for consensus discussions which are ongoing. We have amended our wording as suggested. We have also added a line in the introduction to highlight that existence of these consensus discussions [page 8 line 135-142]. We hope this systematic review is useful with respect to the publication of International consensus guidelines next year. [page 19 line 395-396]

7. Line 324: The word 'must' is inappropriate. For most people and most doctors, HbA1c provides a practical and appropriate basis for definition. However, haemoglobinopathies, iron deficiency states and pregnancy make it likely that an either or approach will be necessary.

> **Response.** We have rewritten this paragraph to try and communicate a more nuanced approach. We try and anticipate potential problems with using HbA1c OR FPG OR 2-hr PG whilst still being mindful that this is likely still the best compromise. [Page 19 line 379-384]

Reviewer 2
I congratulate the Authors for this interesting work. In my opinion, this qualitative review about the definition of "remission" of type 2 diabetes has no methodological limitations and intercepts an important problem. Lack of consensus on definitions is certainly one of the contributing factor of poor research reproducibility. Very good the choice of using Sankey plots.

I have two major comments and two minor:

1. In this paper, the Authors have the opportunity to highlight a chronic problem in the nosology of diseases: the over-tendency of categorising continuous conditions. Beyond the well-documented disastrous effects of this decision on quantitative investigations (classical examples are actually HbA1c/FPG/OGTT groups as well as blood pressure or eGFR groups), here the Authors have the occasion to discuss/comment on the relevance of defining diabetes beyond certain thresholds of biochemical parameters (and hence, remission below the thresholds). The decision of categorising everything has resulted, for example, in an exceptional expansion of "medical diseases" from ICD-10 to ICD-11 (which, for example, includes also "pre-diabetes"). "Remission of diabetes" is just another sub-product of this tendency, as clearly demonstrated by the findings of this systematic review. These three articles, among others, may be helpful in this discussion: Ann Intern Med. 2008; 149:200-3; N Engl J Med 2018; 379:1452-1462; N Engl J Med 2020; 382:1273-1277.
In a patient on insulin therapy with HbA1c of 11%, an intervention that results in a HbA1c of 7.1%, and replacement of insulin with metformin is by far more beneficial than a "remission" from HbA1 of 6.6% with no medication to 5.9% with no medication. Both large observational studies and experimental evidence indicate a continuous benefit for lower HbA1c values, certainly for microvascular and possibly for macrovascular diseases.
In short, I would suggest to discuss more about the appropriateness/importance/necessity of defining a "remission" and about the existence itself of a "remission".

> **Response.** We have read the articles with interest and spent time considering the shifting diagnostic criteria of type 2 diabetes over the last few decades. We agree that mentioning the categorisation of a continuous variable is valuable and enhances the discussion. Therefore, we have restructured our paragraph on implications for research to add more details about the classification of diseases such as diabetes and how this relates to the fundamental (and persistent) problem of defining remission of type 2 diabetes [page 18 line 345-356]. We have also included this as a point in our author summary [page 5 line 75-78] We have broadened the scope of the discussion by introducing the possibility of side-stepping remission and focusing on the development of risk prediction models which as you suggest is a totally different but valid approach that should be highlighted. [Page 18 line 360-363]

2. The paper should be re-checked: some tables in the supplement are not mentioned in the main test; the order of the tables is not correct (first supplemental table quoted in the text is S5; first table in the supplement should be S1 not S3). A thoughtful proofreading (particularly the discussion paragraph) is necessary to improve the readability and quality of the paper.

> **Response.** We have fixed the table order to match the order cited in main text. The whole manuscript has been proofread, the discussion in particular has been restructured and proofread by all authors.

Minor:
1. The PRISMA checklist should be reported in the Supplement.

   Response. We have submitted a PRISMA checklist along with the submission and within the supplementary material.

2. In a list, please separate elements with ";" not with "."

> **Response.** We have checked all lists and changed the separation of elements.

Reviewer 3:

Points of detail

Page 7 Usually I would raise an objection to the exclusion of material not published in English. In this case though I take the purpose of the authors' efforts not to be a complete summary of the entirety of world literature but more an attempt to justify the message: 'Guys, this is a mess. Get a grip.'. In that context they

have found enough and finding that Brazilian Portuguese articles use yet another handful of definitions is hardly going to matter.

Page 8 Was the data extraction done in duplicate too? It seems from the description that this was probably not as trivial a task as it is often, wrongly, assumed to be.

> **Response.** All elements of screening and extracting were done independently and in duplicate by two reviewers. We have added this detail to the methods [page 10 line 174-175] and to the strengths and limitations paragraph in our discussion. Page 17 [line 317]

Page 11 'multiply [. . .] by 18 to calculate'. While this is true there are not that many here and providing both forms would seem kind. I agree that some of the supplementary tables would become inelegant if every single instance was converted.

> **Response.** We have made these changes as suggested throughout the manuscript and in the main tables.

Page 13, Figure 2 I am afraid that as they reached me neither the pdf nor the tiff was legible even when zoomed in. Since these are more akin to what Tukey termed archival graphics rather than impact graphics we probably do not need them all in one figure to enable comparison so making four separate figures might help legibility unless there is some other technical fix. Figure S3 has the same issue.

> **Response.** Apologies, I am not sure what went wrong in the conversion to the PDF manuscript. We have provided each Sankey diagram separately to aid legibility (and also included one figure combining the four Sankey plots just in case for figure 2 and can also do so for figure S3 (now S1). The plots are provided in tiff format and are legible with good resolution on downloading. The best resolution is with a scalable vector graphic format which can be provided if needed.

Page 15 For me the prefix pre- means before so I would find the concept of remission of pre-diabetes to mean returning from the state of pre- diabetes without ever having gone past it to Type 2 diabetes. If you are transitioning back from Type 2 then surely you are in post-diabetes. I suspect this argument will run and run.

> **Response.** We agree that attempting to define remission of pre-diabetes is a problem that is probably avoided (or at least delayed). We have presented the likely complexities in trying to arrive at a definition for remission of prediabetes (analogous to complete remission) in table 4, but have added clarification in the discussion that aiming to define remission of prediabetes would be highly complex. [page 18 line 358-360]

Summary

I think the authors' list of limitations is a fair one but my personal view is that they are not a concern since, as I mentioned above under language, this is not the conventional systematic review and we should not judge it by conventional methods.

> **Response.** Many thanks, as you say, this is not a conventional systematic review. We hope that by taking a different approach we have been able to highlight issues that are important but beyond the scope of previous systematic reviews and meta-analyses that have focussed on a remission outcome.
