# Supplementary material for: Defining remission of type 2 diabetes in research studies: A systematic scoping review
Source: PLoS Med. 2020 Oct 28;17(10):e1003396. doi: 10.1371/journal.pmed.1003396 (PMC7592769; doi:10.1371/journal.pmed.1003396)
Supplement: Attachment — Submitted filename: Response to reviewers_2.docx [file pmed.1003396.s013.docx]

Dr Artur Arikainen

Associate Editor

Plos Medicine

26^th^ August 2020

Dear Dr Arikainen,

**Defining remission of type 2 diabetes in research studies: A systematic scoping review**

Many thanks for your provisional acceptance letter and comments. We have responded to the minor corrections. For your convenience we reproduce the editorial comments verbatim, before detailing our response to each of the points made. Page and line numbers refer to the clean manuscript revision.

We hope our revised manuscript now meets with your approval. This manuscript has been seen and approved by each author. Please feel free to contact me if you require any further clarifications.

Your Sincerely,

Dr Mireille Captieux, on behalf of all authors.

**Response to editors’ comments:**

1.	Abstract:

a.	Please summarise the numbers of each study type included (observational vs. interventional), and by regions or countries.

**Response** We have summarised the numbers of studies by their design and added the number of countries included by our studies within the abstract (line 31-32).

b.	Please include a summary of inclusion criteria, eg. studies in the English language, with 100 or more participants.

**Response** We have included a summary of our inclusion criteria within the abstract (line 27-29).

c.	Line 40: Please include percentage: “(47 definitions, 20%)”.

**Response** We have rectified this omission (line 36).

d.	Please remove this sentence: “The direction of any bias is towards underestimating heterogeneity in defining remission.”

**Response** We have removed this sentence as suggested.

e.	Please add one or two more limitations to the end of the Methods and Findings subsection.

**Response** We have two more limitations to the Methods and Findings section of the abstract (line 38-39)

f.	Line 45: Begin with “We found that…”

**Response** This has been added in (line 41)

g.	Please include the principal source of funding.

**Response** We have added the principal source of funding (MC’s CSO grant). Details of the grant number were given on the online submission form (line 57-58). The publications team have subsequently told us to remove the financial disclosure.

h.	Please move the registration information from lines 82-83 here.

**Response** The registration information has been moved as suggested (line 48-49).

2.	Please remove spaces from within citation callouts, eg. “…and an ageing population [2,3].”, but keep the space between the text and the callout itself as is.

**Response** The spaces within the citation callouts have been removed throughout the manuscript.

3.	Introduction: Please mention briefly the position of the International Diabetes Federation on remission definition(s), if any.

**Response** Thank you for this suggestion, we did find a reference to remission in their 2017 primary care guidelines so have summarised their comment in the introduction (136-139).

4.	Line 168: Please include day for the start date.

**Response** This has now been added (line 167)

5.	Fig 2 legend: Please replace the colour box with a description (eg. “Red indicates…”).

**Response** We have removed the box and replaced it with the suggested text (line 251).

6.	Please check the URL for reference 16.

**Response** The URL for reference 16 has now been checked and should take the reader to the Diabetes UK interim position statement if copied and pasted into a web browser.

7.	Please remove the duplicate PRISMA checklist from the other Supporting Information file.

**Response** The duplicate PRISMA checklist has been removed from the other Supporting Information file and retained within the supplementary material (S1 Table).

8.	We would also recommend replacing your PRISMA checklist with the version specifically for scoping reviews: <http://www.prisma-statement.org/Extensions/ScopingReviews>

**Response** We have now replaced our PRISMA checklist with the version for scoping reviews and highlighted this in the manuscript text (line 147-149 and line 316-317). As previously advised we have used section and paragraph numbers rather than page numbers.

| **NINE Edinburgh BioQuarter**  **9 Little France Road Edinburgh EH16 4UX** usher.bioquarter@ed.ac.uk | **Old Medical School**  **Teviot Place Edinburgh EH8 9AG** usher.teviot@ed.ac.uk | **www.ed.ac.uk/usher**  The University of Edinburgh is a charitable body, registered in Scotland, with registration number SC005336. |
| --- | --- | --- |
